# Meta-Analyses of Methionine Source Concept Validation Trials in Broilers

**DOI:** 10.3390/ani14121771

**Published:** 2024-06-12

**Authors:** Andreas Lemme, Zeyang Li, Juliano Dorigam

**Affiliations:** Evonik Operations GmbH, 63457 Hanau, Germany

**Keywords:** meta-analysis, methionine sources, broiler, body weight gain, feed conversion ratio

## Abstract

**Simple Summary:**

Methionine (Met) and cysteine (Cys) are usually performance-limiting amino acids in broiler feed. To balance diets in order to meet birds’ requirements, DL-Methionine (DL-Met) or the hydroxy analogue of methionine (MHA), which is a precursor of methionine, are common supplements. Based on a multitude of simultaneous dose–response trials, a 65% bioefficacy is recommended for MHA relative to DL-Met. This recommendation has often been tested under various experimental conditions. The present meta-analysis of effect sizes from 95 data sets revealed very little heterogeneity of data combined with the small effect sizes of DL-Met treatments when DL-Met replaced MHA at a weight ratio of 65:100. The effect sizes of DL-Met treatments were not different from MHA treatments, which confirms that 100 units of an MHA product can be replaced by 65 units of DL-Met without compromising performance. The data analysis also revealed that this result was not affected by the broiler strain, the diet type, or the MHA product type being used in the feeding experiments. Moreover, the broiler feeding trials supposed to challenge the 65% bioefficacy recommendation were successful at any dietary Met+Cys supply, including the most sensitive test conditions at marginal Met+Cys supply.

**Abstract:**

While the supplementation of methionine (Met) sources in broiler feeds has been established for several decades, there is debate on the nutritional value of the methionine hydroxy analogue of methionine (MHA) relative to DL-Met. Based on a recommendation suggesting that MHA is 65% as effective as DL-Met, many feeding trials have been conducted to challenge this recommendation. A literature search found 25 publications contributing 95 data sets suitable to compute Hedges’ g effect sizes used in the meta-analysis. The data had very little heterogeneity of almost zero and the small effect sizes of the DL-Met results were not significantly different from MHA. Data were split in various subgroups, finally suggesting that neither broiler strain (Cobb 500, Ross 308), diet type (corn, wheat based), origin of data (peer-reviewed, grey literature), nor MHA product (MHA-free acid, MHA-calcium salt) impacted the outcome of the meta-analysis. Moreover, distinguishing data in groups with dietary Met+Cysteine (Cys) levels below, at, or above requirement demonstrated that there was no interaction with general Met+Cys supply. It is therefore concluded that MHA products can be replaced by DL-Met in a weight-to-weight ratio of 100:65 in any production condition without compromising broiler performance

## 1. Introduction

In 1957, nine years after Werner Schwarze synthesized the first kg of DL-Methionine (**DL-Met**) in Germany, Rosenberg [1] concluded that methionine would be the first limiting amino acid in compound feed. In the early 1950s, Degussa AG produced about 360 t DL-Met per year [2]. Shortly after, the hydroxy analogue of methionine (**MHA**) was developed [3]. Interestingly, the polymers of MHA were considered “not useful as feed components” in a patent on the preparation of methionine analogues [4]. Since then, differences between DL-Met and MHA with respect to their nutritional value have been known. Therefore, the effectiveness of MHA products relative to DL-Met was evaluated in the following years [5,6,7]. Boebel and Baker [8] and van Weerden et al. [9] used slope-ratio assays revealing that MHA-free acid (**MHA-FA**) and calcium salt of MHA (**MHA-Ca**) are, respectively, 66–68% and 67–72% as efficient as DL-Met in broilers when compared on a weight basis (in contrast to an equimolar comparison). Interestingly, Boebel and Baker [8] also reported a considerably lower efficacy of MHA polymers, which was confirmed by others [10,11]. In the 1980s, the effectiveness of MHA relative to DL-Met was investigated by Rhône-Poulenc, who was the early predecessor of Adisseo France SAS and who, in contrast to Adisseo France SAS today, was only producing DL-Met. They used simultaneous or multi-exponential regression for Met source comparison and reported 66% efficacy of MHA-FA relative to DL-Met on a weight basis [12]. They introduced a concept for assessing the nutritional value of MHA by including an internal standard. According to their overall conclusion, MHA-FA was 70% as effective as DL-Met on a weight basis and, therefore, they developed a diluted DL-Met additive with 70% purity [10,13]. Indeed, responses of broilers to diluted DL-Met were similar to the responses obtained with MHA-FA.

Since then, many trials have been conducted and reported, and the discussion around the nutritional value of MHA-FA in particular, which is most prominent among MHA products [2], is ongoing because the nutritional value assigned to MHA relative to DL-Met is decisive for the performance of the animals, which directly affects the income side of broiler production on the one hand and the diet cost on the other. In a compilation of data available until 1995, a relative efficacy of 66% for MHA-FA was concluded [14], which means that 100 parts of MHA-FA in feed can be replaced by 66 parts of DL-Met without compromising animal performance. With a preceding agreement on methodology, Novus international Inc., Aventis Animal Nutrition (today known as Adisseo France SAS), and Degussa AG (today known as Evonik Industries GmbH) participated in a meta-analysis project by the Dutch Centraal Veevoederbureau in 2003 aiming to determine the efficacy of MHA-FA relative to DL-Met in farm animals [15]. The analysis revealed that MHA-FA was 68% as efficient as DL-Met for broilers on a weight basis and, significantly different to the 88% which is the MHA content in MHA-FA. Also, the European Food Safety Authority recently concluded that “there is convincing evidence” that MHA-FA and MHA-Ca have significantly lower bioefficacy than DL-Met in non-ruminant species [16]. In this report, an efficacy of 67% for MHA was indicated. Analogous to the abovementioned approach by Rhône-Poulenc, six experiments were conducted including 65% pure DL-Met (diluted) next to MHA and DL-Met [17,18,19,20]. Those were analyzed in a meta-analysis revealing an average efficacy of 62% and 63% for 65% diluted DL-Met and MHA-FA relative to pure DL-Met, respectively [17]. It should be noted that the bioefficacy of 65% diluted DL-Met was determined by simultaneous regression analysis as proposed by Littell et al. [21]. With that said, the result being very close to the expected 65% validates the methodological approach [17].

Although releasing consistent results, the multi-exponential regression approach has been criticized several times. Understandably, researchers from MHA-producing companies in particular have repeatedly tried to discredit both the methodology and the finally recommended bioefficacy of 65% for MHA. When Vázquez-Añón et al. [22] analyzed the results of four dose–response feeding studies separately for each trial, linear, exponential, and quadratic regressions fit best to DL-Met and MHA-FA responses but were different between trials and products. However, a combined analysis of these four trials suggested a linear response to MHA-FA with no asymptote and a quadratic response for DL-Met [22]. Also, in 2006, the same authors reported quadratic responses for both products [23], whereas a further broiler trial suggested linear responses to both products [24]. Altogether, these publications demonstrate biologically fallacious and inconsistent conclusions but state that there are no efficacy differences between the products. Indeed, the linear regressions by Liu et al. [24] were used for comparing slopes, but there were no significant responses of broilers to either product, which would be a prerequisite for a reliable slope-ratio assay [21]. After an unconventional processing of data, a meta-analysis using the slope-ratio comparison concluded that the efficiency of MHA-FA would not differ from that of DL-Met [25]. The authors of that paper could only partly justify doubts on reliability and scientific adequacy regarding data treatment and the selection of the finally used publications [26,27]. Kratzer, who co-authored [23], and Littell, who originally described the slope-ratio and multi-exponential approach [21], referred to the abovementioned study by Jansman et al. [15] and concluded that response curves to MHA-FA and DL-Met would differ in their asymptote, and therefore, the conclusion of lower bioefficacy of MHA-FA compared to DL-Met would be invalid [28]. While an immediate response to this article denied this statement, a deep-dive meta-analysis then provided clear evidence that asymptotes for both products are similar, while the required dose for either product to approach the asymptote differs [29,30].

More recently, it was argued that a fair comparison should be made at “commercial” supplementation levels at requirement [31]. Batonon-Alavo et al. [32] supplemented DL-Met and MHA-FA at equimolar levels at broiler breeder Met+Cys recommendations and introduced the non-inferiority test as a “new statistical approach” to show that MHA-FA is not inferior to DL-Met. Again, major errors were complained about and the authors only partly responded [33,34]. Interestingly, the non-inferiority test pre-assumes a lower efficacy of the product under test—in this case, MHA-FA—and the question to be solved is just whether this lower efficacy is in an acceptable range. Moreover, a correct application of the method revealed that non-inferiority cannot be confirmed for MHA-FA compared to DL-Met [34].

In summary, the previous paragraphs indicate a controversial discussion on the bioefficacy of methionine sources but also show the aim to create doubt on the correctness and validity of the multi-regression approach for the determination of a product’s efficacy relative to another product [22,23,24,25,28,32]. In order to provide further evidence on the seriousness, validity, and applicability of the concluded bioefficacy of 65% for MHA on a weight basis relative to DL-Met [17,35,36], a meta-analysis was conducted using Hedges’ g effect size. This analysis utilized data from broiler feeding studies in which we applied the recommended relative bioefficacy by comparing MHA treatments with a DL-Met treatment where the DL-Met supplementation was 65% as high as that of MHA. Moreover, the methodology allows us to consider further experimental conditions and their impact on the outcome and, thus, offers the opportunity to substantiate evidence that a bioefficacy of 65% for MHA is applicable under any conditions. Among the latter, the question as to whether the general dietary Met+Cysteine (Cys) level interacts with the applicability of the recommendation is addressed.

## 2. Materials and Methods

### 2.1. Data Collection and Screening

**Figure 1:**
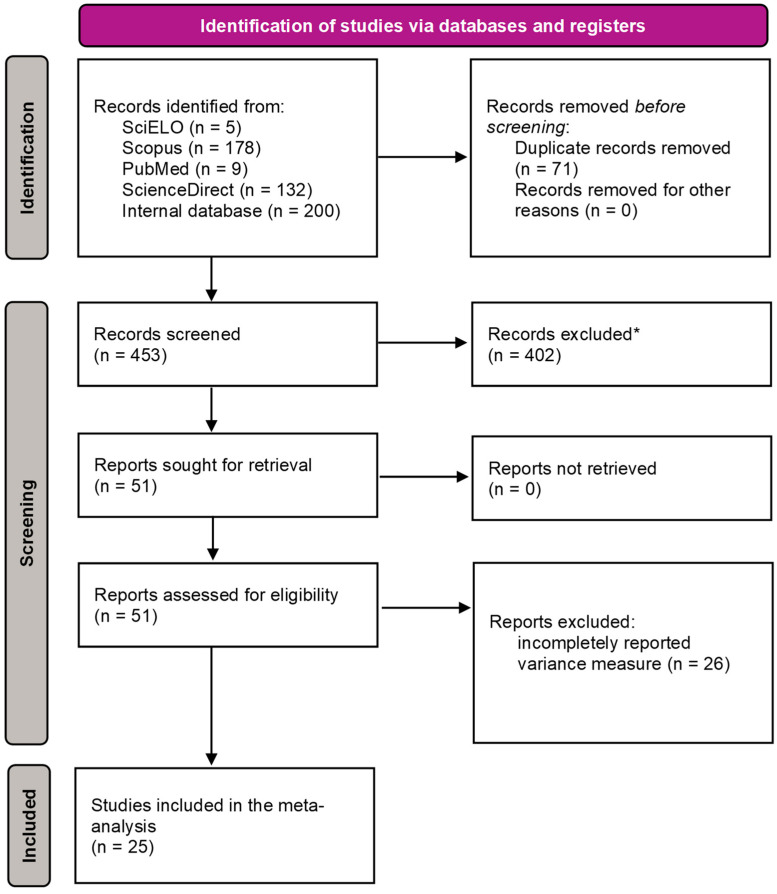
Prisma flow diagram showing the selection procedure for broiler feeding studies with number (n) of publications identified, screened, and selected for the meta-analysis. * Studies that do not have treatment with DL-Met dosed 65% as much as MHA, studies that did not use broilers, and studies that did not provide information on dietary Met+Cys levels or that do not allow for recalculation of dietary Met+Cys level were excluded.

The studies examined were retrieved from public databases (SciELO, Scopus, PubMed and Science Direct) and complemented with an internal database, which contains publications dating from 2002 to 2023. The studies considered the use of DL-Met and MHA tested in the weight-to-weight proportion of 65:100 in broiler feeds. The following keywords and query strings were used: (DL-Methionine) AND (DL-HMBA OR DL-HMTBA OR MHA) AND (broilers). The criteria of inclusion in the meta-analysis were that (1) the study specified a treatment group with DL-Met which is 65% as much dosed as the corresponding equivalent of MHA, and an MHA treatment; (2) the dietary Met+Cys was informed or could be recalculated; (3) information was provided about the study design (breed, sex, age, number of birds, replicates per treatment); (4) the measure of variability was appropriate (coefficient of variation, standard deviation, standard error, standard error of the mean, etc.); (5) information about feed composition was provided; and (6) the feed conversion ratio and weight gain were provided. On this basis, 25 publications were included (Figure 1).

The details of the included studies are shown in Table 1. Out of the 524 publications initially retrieved from the databases, 71 were excluded due to duplication, leaving 453 potentially relevant studies. After applying our inclusion criteria, 402 studies were further excluded, leaving 51 studies for full-text analysis. Of these, 26 studies did not report a measure of variability and were excluded from the final analysis. The remaining 25 studies were included in our meta-analysis.

### 2.2. Data Collection

The mean values, standard deviations (SDs), and sample sizes (n, representing the number of replicates) were extracted from each included study. The target variables in this study were body weight gain (BWG) and the feed conversion ratio (FCR). When a study used the standard error of means (SEM) or coefficient of variation (CV) as a variance measurement, the SD values were calculated according to the following equations:

SD = SEM × √n(1)

SD = CV × Mean(2)

When the least significant difference (LSD) was provided, the EX-TRACT tool was used to estimate the standard deviations from published articles [59]. The experiments were encoded independently, each including two dietary treatments (MHA-FA or MHA-Ca and DLM65, DL-Met being dosed 65% as high as MHA-FA or MHA-Ca in corresponding treatments on a product weight basis). Studies with results for more than one dietary Met+Cys level were assigned as below, at, or above requirement. AMINOChick^®^ 3.0 software [60] was used as reference for Met+Cys recommendations for the respective trial periods. The proportions of each dietary Met+Cys level relative to the recommended dietary Met+Cys level were used to assign the treatments into the respective groups. In additional groupings, studies were also assigned to either different MHA product subgroups (MHA-FA; MHA-Ca), diet type subgroups (corn; wheat; corn–wheat combination), or broiler strain subgroups (Cobb500; Ross308) to evaluate if any of these factors impacted broiler performance in the selected studies. Additionally, studies were grouped into those obtained from peer-reviewed papers and those obtained from publications which were not peer-reviewed to examine whether the source of information impacted the overall conclusion of this meta-analysis. The latter subgroup include data available from conference abstracts as well as Evonik-specific trial reports (Facts&Figures).

### 2.3. Data Analysis

Data analysis was performed using Meta-Essential version 1.5 [61]. The estimated effect size (the difference in performance between DLM65 and MHA as reference groups divided by the pooled SD) was quantified and bias corrected using Hedges’ g with a 95% confidence interval (CI) [62]. The effect sizes were considered as no effect (0.0), small (0.2), medium (0.5), or large (0.8) based on benchmarks suggested by Cohen [63]. Data were pooled using a fixed-effect model in case of the absence of heterogeneity (*p* > 0.05) and after being pre-checked using the I^2^ value [64]. Treatment effects were considered not statistically significant when the CI included zero; otherwise, they were considered significantly positive when the CIs were positive but excluded zero or significantly negative when the CIs were negative but excluded zero.

Moreover, outlier and influential cases were examined when conducting the meta-analysis and removed accordingly [65]. A publication bias evaluation was also carried out using visual assessment with a funnel plot and the ‘trim and fill’ method to identify asymmetry arising from publication bias [66]. Additional statistical tests were also used to quantify publication bias, such as Begg’s rank test [67] and Egger’s regression test [68]. Begg’s rank test examines the rank correlation (Kendall’s Tau) between the ranks of effect sizes and the corresponding ranks of their variances, suggesting that a strong correlation implies publication bias. Egger’s regression test regresses the standardized effect sizes of their precisions, where the regression intercept is expected to be zero in the absence of publication bias.

Effect sizes and upper and lower limits of CI are reported for individual treatment pairs in the result tables. Heterogeneity and respective *p*-values as well as effect size and respective CIs are reported for respective subgroups and combined overall including all data across subgroups.

## 3. Results and Discussion

The evaluation of these data can be ascribed to the long-lasting debate on the nutritional value of MHA compared to DL-Met. While comparative regression analysis was applied already in the early 1980s [8,9,69], not only the methodology behind the multi-exponential regression has been validated [17,18,19,20,30,35], but also, a recommendation for application has been derived [17,35]. Accordingly, MHA can be replaced by DL-Met in a weight ratio of 100:65 without compromising animal performance. This recommendation has been challenged in many trials, and those which were suitable for a meta-analysis according to the selection process described in Figure 1 were compiled and analyzed. A total of 95 data sets out of 25 publications were available for the meta-analysis (Table 1).

### 3.1. Publication Bias

The removal of extreme effect sizes resulted in the absence of heterogeneity with respect to combined effect sizes for BWG (I^2^ = 0.00%, *p* = 0.578 (broiler strain differentiation) to *p* = 0.835 (dietary Met+Cys level differentiation)) and FCR (I^2^ = 0.00%, *p* = 0.491 (dietary Met+Cys level differentiation) to *p* = 0.850 (broiler strain differentiation)) data in the five meta-analyses. Therefore, the data sets were highly homogenous with very little variation.

**Figure 2:**
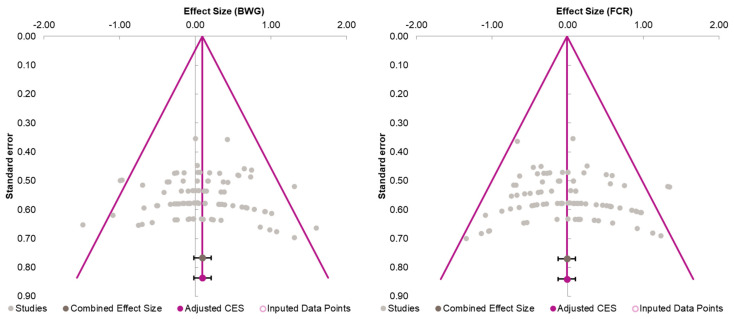
Funnel plot showing the standard error plotted against the combined effect size. Adjusted combined effect size (CES) recalculates the combined effect size considering inputted data points when they exist. Graphs automatically generated by Meta-Essential version 1.5 [61].

Moreover, the absence of imputed data points (based on the trim and fill method) in the funnel plot indicates that there is no asymmetry in the distribution of effect sizes. Figure 2 serves as a funnel plot example showing the results of the evaluation with dietary Met+Cys subgroups. In addition, other bias measurements such as Begg–Mazumdar’s rank correlation test (Kendall’s tau for BWG = 0.03 with *p* = 0.362 and for FCR = 0.11 with *p* = 0.062) and Egger’s regression intercept for BWG = 0.02 (95% CI = −1.40 to 1.45, *p* = 0.973) and FCR = 0.86 (95% CI = −0.66 to 2.38, *p* = 0.264) indicate the absence of bias by publication for this evaluation. Absence of bias by publication was also identified for the meta-analyses where data were subgrouped for MHA products, publication type, diet type or, broiler strains (Table 2).

### 3.2. Methionine+Cysteine Specification Setting in Experimental Diets

As shown in Table 3, no significant heterogeneity was found for BWG when the methionine sources were supplemented above (I^2^ = 9.7%, *p* = 0.35), at (I^2^ = 0.0%, *p* = 0.84), or below (I^2^ = 0.0%, *p* = 0.87) dietary Met+Cys recommendation. A medium positive effect size of 0.28 in favour of DLM65 was observed in the subgroup “at requirement” as the CI excluded zero (0.08 to 0.48). While a similar but insignificant effect size for “above requirement” was computed, the effect size was almost zero for “below requirement”. Analogous to BWG data, no significant heterogeneity was found for FCR in either subgroup (Table 4). With values of 0.02, −0.08, and 0.01, the effect sizes of the respective subgroups were small and not significantly different from zero. These results indicate that the BWG and FCR of the broilers of the corresponding MHA–DLM65 treatments were similar and not different. From this overall perspective, it can be concluded that the recommended bioefficacy of 65% for MHA is applicable at any dietary Met+Cys supply status without any risk of compromising performance.

Dietary Met+Cys levels in poultry feeds are commonly not sufficient to meet the birds’ requirements. Therefore, supplementation of methionine sources has been established for more than 50 years [2]. The gradual addition of methionine to a diet deficient in total sulphur amino acids (TSAAs) typically results in a non-linear and asymptotic improvement in performance [70], which implies that increasing supplementation above the level where the response curve approximates the asymptote has no further impact on performance and is thus an over-supply. Only severe over-supply would result in performance depressions and limit the asymptotic plateau [70,71]. This is important insofar as Met+Cys settings under commercial feeding conditions are often slightly above requirements, that is, in the early part of the asymptote. Safety margins shall avoid marginal supply because of Met+Cys variation in feed ingredients, but also because of the changing Met+Cys requirements of animals. For instance, it has recently been shown that stressors such as heat stress, stocking density, or pathogens can increase the metabolic demand for methionine [72,73,74]. If the supplementation of methionine sources operates in such commercial over-supply conditions, it might be easy to demonstrate both a replacement of MHA by DL-Met in a 100:65 ratio [17,35] and a replacement of DL-Met by MHA in a 88:100 ratio, as suggested by others [23,25,32]. For example, Batonon-Alavo et al. [32] purposely supplemented DL-Met and MHA-FA in a ratio of 88:100 at doses to meet recommended TSAA levels for broilers and reported no difference. In another trial, doses of products at an 88:100 ratio should have been 10% below, at, or 10% above requirement, but responses clearly indicated that the asymptote was already achieved with the lower dose [31]. Indeed, a large-scale experiment including 26 broiler farms receiving feed with MHA-FA and 24 broiler farms receiving feed with DL-Met (supplemented at 88% of the corresponding MHA-FA volume) revealed the same performance for both products [75]. Other researchers even concluded that MHA would outperform DL-Met, especially above the Met+Cys requirement, because of potential additional effects [22,31]. The meta-analysis of subgroups at or above requirement (Table 3 and Table 4) showed similar BWGs or FCRs. Thus, an outperforming effect of MHA over DL-Met cannot be confirmed.

Nutritionists cannot derive a robust conclusion on the validity of the 65:100 recommendation under those conditions, as more sensitive test conditions would be needed. Such were achieved by challenging the recommended 65% bioefficacy for MHA at a suboptimal dietary Met+Cys supply. Indeed, the meta-analysis for the subgroup “below” requirement provides evidence that the recommendation is successfully applicable, therefore validating the recommendation (Table 3 and Table 4). Moreover, while the Hedges’ g only includes pairs of corresponding treatments, it is noteworthy that respective individual publications provide evidence that Met+Cys levels were in fact below requirement because they exhibited significantly lower performance compared to other treatments with higher supplementation levels. These other treatments are included in the subgroups “at” or “above requirement”. Batonon-Alavo and Rouffineau suggested that Met source comparison trials should be run at commercial level (see previous paragraph [32]) but compared DL-Met and MHA-FA trial data only below the requirement in another study while excluding all data above the requirement [25]. In that meta-analysis, the growth responses were regressed against methionine intake above basal diets (as well as Met+Cys levels above basal diet, which does not make sense in a Met source comparison [26]), but they did not share information as to what methionine value they assigned to MHA-FA. Therefore, the final conclusion that the slopes for the two products did not differ is questionable because a certain (high?) assumed bioefficacy of MHA-FA autocorrelated with the final outcome. The strong statistics behind the current subset “below requirement” strongly implies an applicable bioefficacy of 65% for MHA-FA.

The outcome of the meta-analyses is confirmed by similar research on turkey nutrition [36]. That literature survey indicated the same performance of turkeys fed MHA or DL-Met supplemented at a 100:65 weight ratio, both at marginal supply and at the requirement level [36]. This validation of the concept also held true for meat yield in turkeys, being a very sensitive parameter for amino acid nutrition [76]. While not considered in the current meta-analyses, some of the included studies also reported the same breast meat yield for corresponding MHA and DLM65 treatments at low, adequate, and high TSAA levels [17,18,35].

### 3.3. Two MHA Products

Data were divided into subgroups based on the type of MHA product, specifically into those where DLM65 was compared to MHA-FA and those where DLM65 was compared to MHA-Ca. Respective meta-analyses for BWG and FCR results are reported in Table 5 and Table 6. For BWG data, no significant heterogeneity was found for either MHA-FA (I^2^ = 7.7%, *p* = 0.30) or for the MHA-Ca subgroup (I^2^ = 0.0%, *p* = 1.00). A small positive but insignificant effect size of 0.14 in favour of DLM65 was observed in subgroup MHA-FA with respect to BWG, whilst the effect size in subgroup MHA-Ca was zero (Table 5). Interestingly, within the MHA-FA subgroup, there was one data set with a large and significant positive effect size indicating that DLM65 outperformed MHA-FA. On the other hand, there was one data set where MHA-FA-fed broilers had a significantly lower FCR effect size than DLM65. However, this was an exception, as the overall effect sizes for the MHA-Ca and MHA-FA subgroups were not significant at −0.1 and zero, respectively (Table 6). Based on the meta-analyses of the MHA product subgroups, it is confirmed that replacing both MHA-FA and MHA-Ca with DL-Met at a 65% weight ratio did not have any detrimental effect on performance. This suggests that exactly the same concept can be applied for both MHA products. This is interesting insofar as MHA products differ in their chemical composition. Liquid MHA-FA consists of 12% water, 65% MHA monomers, 18% MHA dimers, 3% MHA trimers and 2% higher MHA oligomers [77,78,79], whereas MHA-Ca consists of about 84% monomeric MHA and at least 12% calcium [16]. In the very early years of MHA-FA production, a nutritional disadvantage has been reported particularly for the oligomers of MHA [4,8,10,11]. Indeed, later, it could be shown that the dimers and trimers in particular are almost entirely not absorbed, as demonstrated using an in vivo broiler model [80]. Still, the monomeric MHA-Ca was also less efficiently absorbed and utilized by broilers [81,82]. So, while absorption mechanisms [83] and interactions with intestinal microbiota [84,85,86] generally interact with MHA availability, the lower concentration of MHA in the MHA-Ca product counterbalance the better availability of the monomers. Consequently, a very similar bioefficacy of MHA-Ca and MHA-FA relative to DL-Met was determined on a product weight basis [17,35]. The similar outcome for both subgroups in the current meta-analysis is, thus, not surprising.

### 3.4. Source of Data

For meta-analyses, information needs to be compiled. One major source of information is the scientific peer-reviewed literature, as the review process suggests the proven quality of the research. However, other publications, the so-called grey literature, also contribute to the understanding of, e.g., nutritional principles. In order to utilize all available information, holo-analysis has been introduced [87,88], which would include information from any kind of publication. However, meta-analyses on methionine sources also included unpublished reports next to peer-reviewed publications [23], with the effect of such unpublished reports on the outcomes of that meta-analysis not having been examined. In the current meta-analysis, the peer-reviewed and grey literature were included, although all are publicly available. As can be seen in Table 7 and Table 8, the number of data sets from both publication types was well balanced. No heterogeneity was found for either publication type for both BWG and FCR data (I^2^ = 0.0%). The respective *p*-values ranged between 0.57 and 0.82 and did not indicate statistical significance. The same applies for the respective combined effect sizes. The effect sizes were small and not significant, which overall suggests that there was no difference due to publication type. It might be argued that the grey literature did not include references from other companies or institutions besides Evonik. Indeed, other companies also conduct feeding trials and publish in their own media, but no experiments in which MHA was replaced by DL-Met in a 100:65 weight ratio could be found.

### 3.5. Broiler Strain

Various broiler genetics were used in the feeding experiments included in the meta-analysis. However, two strains, namely Cobb 500 and Ross 308, dominated, while the other strains were used to a much lesser extent, which did not allow reliable subgroups to be built. Therefore, a total of 73 and 70 data sets could be used for BWG and FCR analyses (Table 9 and Table 10). Interestingly, the combined effect size was small (0.08) but significantly different from zero for BWG, indicating that DLM65 resulted in higher BWG than MHA products (Table 9). However, while the effect sizes for Cobb 500 and Ross 308 subgroups were 0.09 and 0.07, respectively, they were both not different from zero. One single Cobb 500 data set showed a significantly lower effect size of DLM65 compared to MHA, while another Ross 308 data set was significantly higher. With respect to FCR, the effect sizes were small and not significant for subgroups but also as a combined effect size (Table 10). For all subgroups, heterogeneity was low (I^2^ = 0.0 to 8.3%) and not significant (*p* = 0.33 to 0.79).

Although other meta-analyses recorded various broiler strains (e.g., [22,25]), the discussion of the impact of broiler genetics on the efficacy of methionine sources was never a topic in the scientific literature. Insofar, this analysis is additional new information that there is no interaction between genetics and the efficacy of MHA products compared to DLM65. However, even beyond broiler strains, the concept of 65% relative efficacy for MHA is successful for animals with other genetic bases. A recent compilation of both dose–response and 65:100 concept challenge trials with turkeys revealed the same performance of turkeys fed with DLM65 compared to turkeys fed with MHA-FA [36]. Also, in laying hens, MHA-FA could successfully be replaced by 65% DL-Met in commercial conditions [89]. This proves the opposite of the statement by Vázquez-Añón et al. [90], who concluded in their review that MHA-FA would have “full bioefficacy” and that “there were no differences between the two methionine sources” in laying hen and turkey nutrition. Although Souza et al. [91] concluded in their review on methionine sources in piglets that the efficacy of equimolar levels of DL-Met and MHA would not differ at or below Met requirement, there are several studies with pigs showing that 65:100 replacement results in the same performance and that this holds true below and at Met requirement [92,93,94,95]. It is therefore concluded that the concept of MHA replacement by DL-Met in a 100:65 weight ratio can be implemented in the nutrition of any farm species without compromising performance.

### 3.6. Diet Type

While broiler feed composition can vary widely, ingredients can influence digestion and nutrient availability. Although formulated for the same energy and digestible amino acid concentrations, corn-based diets revealed significantly higher AMEn than wheat-based diets in cases where wheat was ground and not added unground [96]. Indeed, starch digestibility was significantly affected while protein digestibility was not, despite the apparent digestibility of a few amino acids being significantly reduced with wheat. This was not confirmed by Ghayour-Najafabadi et al. [97], but the jejunal villus length was higher with wheat-based diets. Others reported a significantly higher phylogenetic diversity in the cecal microbiome of corn-fed broilers in contrast to wheat-fed broilers [98]. Interestingly, a lower intestinal pH was reported for wheat-fed broilers compared to corn-fed broilers [99], which might then interact with MHA-FA metabolism, as this is an organic acid with a pH of 1, from a chemical point of view. For example, higher acetate, propionate, and butyrate concentrations were reported for MHA-FA-fed broilers, although the pH was not reported [100]. The question as to whether MHA-FA would have added value beyond being a precursor of methionine is controversially discussed. However, any kind of effect would be included in animal performance reported from dose-response studies performed under a wide range of different conditions and which were based on the 65% bioefficacy concept [15,17].

In the current meta-analysis, three subgroups could be distinguished (Table 11 and Table 12). Accordingly, the majority of data sets were based on corn–soybean meal-based diets (n = 60), while 15 data sets refer to corn–wheat–soybean meal and 8 data sets to wheat–soybean meal-based diets. The heterogeneity of effect sizes of the corn and wheat subgroups was not significant and was 0% for both BWG and FCR, which also applies for combined effect sizes. The effect size heterogeneity for the corn–wheat–soybean meal subgroup was 23.2% (*p* = 0.197) for BWG but was 0% for FCR. The effect sizes were small and not different from zero, which overall indicates that the type of diet did not interact with the outcome of feeding trials testing DLM65 against MHA products. Vázquez-Añón et al. [22] reported four simultaneous dose–response broiler trials in which DL-Met or MHA-FA were added in graded levels and which differed in the grain used. Either sorghum, wheat, corn, or corn + meat and bone meal were used, and while responses of a different nature, i.e., linear, quadratic, or exponential, were identified, this observation was not related to grain type. From the present data analysis, it is concluded that MHA products can be replaced by DL-Met at a weight ratio of 100:65 in any kind of diet without compromising broiler performance.

## 4. Conclusions

The purpose of this data analysis was to evaluate whether DL-Met can replace MHA products when added at 65% the amount of MHA. A literature search was conducted and 25 publications providing 95 suitable data sets were found. In this meta-analysis, effect sizes for each comparison between DLM65 and MHA were computed with respect to BWG and FCR. Moreover, data were split into subgroups which distinguished between dietary Met+Cys specifications (below, at, or above requirement), MHA products (MHA-FA, MHA-Ca), type of publication (peer-reviewed, grey literature), broiler strains (Cobb 500, Ross 308) or diet types (corn, corn–wheat, wheat-based). For all analyses, very low heterogeneity, in most cases a heterogeneity of zero, was found combined with small insignificant effect sizes, which means that the performance of DLM65-fed broilers was always not different to MHA-fed broilers. It is therefore concluded that MHA can successfully be replaced by DL-Met dosed just 65% as high as MHA on a product weight basis under any production condition without compromising broiler performance. While this applies for both MHA-FA and MHA-Ca, it is pronounced that the concept can successfully be applied at dietary Met+Cys specifications below requirement which are considered particularly sensitive to such a test.

## Figures and Tables

**Table 1:** Details of the included studies.

Paper	n° Birds Used per Treatment	Sex	Breed	Diet Type ^5^	Period (d)	MHA Used	Avg. Basal Met+Cys Level (%)	Supplementation Level, %	
								65 DLM ^1^	MHA ^2^
Zhang et al. (2017) ^4^ [37]	60	Male	Cobb500	Wheat SBM	10–26	MHA-FA	0.648	0.220	0.340
[38]	168	Male	Cobb500	Corn SBM	0–21	MHA-Ca	0.580	0.074	0.114
[38]	168	Male	Cobb500	Corn SBM	0–21	MHA-Ca	0.580	0.148	0.228
[38]	168	Male	Cobb500	Corn SBM	0–21	MHA-Ca	0.580	0.222	0.342
[38]	168	Male	Cobb500	Corn SBM	0–21	MHA-Ca	0.580	0.296	0.456
[38]	168	Male	Cobb500	Corn SBM	0–21	MHA-Ca	0.580	0.370	0.570
[38]	154	Male	Cobb500	Corn SBM	22–42	MHA-Ca	0.520	0.062	0.095
[38]	154	Male	Cobb500	Corn SBM	22–42	MHA-Ca	0.520	0.124	0.190
[38]	154	Male	Cobb500	Corn SBM	22–42	MHA-Ca	0.520	0.186	0.286
[38]	154	Male	Cobb500	Corn SBM	22–42	MHA-Ca	0.520	0.248	0.381
[38]	154	Male	Cobb500	Corn SBM	22–42	MHA-Ca	0.520	0.310	0.476
[39]	60	Mixed	Ross308	Corn SBM	0–35	MHA-Ca	0.592	0.236	0.364
[40]	220	Male	Cobb400	Corn SBM	0–42	MHA-Ca	0.580	0.255	0.390
[41] ^3^	150	Mixed	Cobb500	Wheat Corn SBM	0–36	MHA-Ca	0.591	0.238	0.366
[42]	144	Male	Ross308	Wheat Corn SBM	0–42	MHA-Ca	0.566	0.255	0.393
[43]	200	Mixed	Cobb400	Corn SBM	0–42	MHA-Ca	0.555	0.223	0.341
[35]	120	Male	Ross308	Wheat Corn SBM	0–38	MHA-Ca	0.568	0.020	0.030
[35]	120	Male	Ross308	Wheat Corn SBM	0–38	MHA-Ca	0.568	0.039	0.060
[35]	120	Male	Ross308	Wheat Corn SBM	0–38	MHA-Ca	0.568	0.065	0.100
[35]	120	Male	Ross308	Wheat Corn SBM	0–38	MHA-Ca	0.568	0.098	0.150
[35]	120	Male	Ross308	Wheat Corn SBM	0–38	MHA-Ca	0.568	0.137	0.210
[19]	180	Male	Ross208	Wheat SBM	0–42	MHA-FA	0.555	0.026	0.040
[19]	180	Male	Ross208	Wheat SBM	0–42	MHA-FA	0.555	0.052	0.080
[19]	180	Male	Ross208	Wheat SBM	0–42	MHA-FA	0.555	0.078	0.120
[19]	180	Male	Ross208	Wheat SBM	0–42	MHA-FA	0.555	0.104	0.160
[19]	180	Male	Ross208	Wheat SBM	0–42	MHA-FA	0.555	0.130	0.200
[19]	360	Male	Hybro	Sorghum SBM	0–42	MHA-FA	0.505	0.039	0.060
[19]	360	Male	Hybro	Sorghum SBM	0–42	MHA-FA	0.505	0.078	0.120
[19]	360	Male	Hybro	Sorghum SBM	0–42	MHA-FA	0.505	0.117	0.180
[19]	360	Male	Hybro	Sorghum SBM	0–42	MHA-FA	0.505	0.156	0.240
[19] ^4^	36	Male	Ross308	Corn SBM	7–35	MHA-FA	0.533	0.020	0.030
[19] ^4^	36	Male	Ross308	Corn SBM	7–35	MHA-FA	0.533	0.039	0.060
[19] ^4^	36	Male	Ross308	Corn SBM	7–35	MHA-FA	0.533	0.059	0.090
[19] ^4^	36	Male	Ross308	Corn SBM	7–35	MHA-FA	0.533	0.078	0.120
[19] ^4^	36	Male	Ross308	Corn SBM	7–35	MHA-FA	0.533	0.098	0.150
[17]	120	Male	Ross308	Wheat Corn SBM	0–35	MHA-FA	0.559	0.026	0.040
[17]	120	Male	Ross308	Wheat Corn SBM	0–35	MHA-FA	0.559	0.052	0.080
[17]	120	Male	Ross308	Wheat Corn SBM	0–35	MHA-FA	0.559	0.078	0.120
[17]	120	Male	Ross308	Wheat Corn SBM	0–35	MHA-FA	0.559	0.137	0.210
[17]	120	Male	Ross308	Wheat Corn SBM	0–35	MHA-FA	0.559	0.195	0.300
[44] ^4^	200	Male	Ross×Ross	Sorghum Wheat SBM	0–42	MHA-FA	0.537	0.039	0.060
[44] ^4^	200	Male	Ross×Ross	Sorghum Wheat SBM	0–42	MHA-FA	0.537	0.078	0.120
[44] ^4^	200	Male	Ross×Ross	Sorghum Wheat SBM	0–42	MHA-FA	0.537	0.117	0.180
[44] ^4^	200	Male	Ross×Ross	Sorghum Wheat SBM	0–42	MHA-FA	0.537	0.156	0.240
[45] ^4^	100	Mixed	Hubbard	Corn SBM	0–42	MHA-FA	0.641	0.180	0.278
[46]	176	Male	Ross308	Corn SBM	0–47	MHA-FA	0.577	0.092	0.136
[46]	176	Male	Ross308	Corn SBM	0–47	MHA-FA	0.577	0.176	0.279
[46]	176	Male	Ross308	Corn SBM	0–47	MHA-FA	0.577	0.269	0.415
[47]	90	Male	Cobb500	Corn SBM	22–42	MHA-FA	0.540	0.093	0.143
[47]	90	Male	Cobb500	Corn SBM	22–42	MHA-FA	0.540	0.186	0.286
[47]	90	Male	Cobb500	Corn SBM	22–42	MHA-FA	0.540	0.279	0.429
[48]	144	Male	Ross308	Wheat SBM	0–35	MHA-FA	0.543	0.086	0.133
[48]	144	Male	Ross308	Wheat SBM	0–35	MHA-FA	0.543	0.249	0.383
[48]	500	Mixed	Ross308	Corn SBM	0–32	MHA-FA	0.545	0.085	0.131
[48]	500	Mixed	Ross308	Corn SBM	0–32	MHA-FA	0.545	0.302	0.464
[49]	128	Mixed	Cobb500	Corn SBM	0–32	MHA-Ca	0.566	0.303	0.466
[50] ^4^	176	Male	Ross308	Corn SBM	0–47	MHA-FA	0.582	0.091	0.139
[50] ^4^	176	Male	Ross308	Corn SBM	0–47	MHA-FA	0.582	0.179	0.276
[50] ^4^	176	Male	Ross308	Corn SBM	0–47	MHA-FA	0.582	0.270	0.415
[51]	216	Male	Cobb500	Corn SBM	21–42	MHA-Ca	0.540	0.056	0.086
[51]	216	Male	Cobb500	Corn SBM	21–42	MHA-Ca	0.540	0.112	0.172
[51]	216	Male	Cobb500	Corn SBM	21–42	MHA-Ca	0.540	0.167	0.258
[51]	216	Male	Cobb500	Corn SBM	21–42	MHA-Ca	0.540	0.223	0.343
[51]	216	Male	Cobb500	Corn SBM	21–42	MHA-Ca	0.540	0.279	0.429
[52]	512	Male	Ross 708	Corn SBM	0–42	MHA-Ca	0.548	0.141	0.218
[52]	512	Male	Ross 708	Corn SBM	0–42	MHA-Ca	0.500	0.179	0.275
[53] ^4^	120	Male	Cobb500	Corn SBM	0–35	MHA-FA	0.502	0.315	0.486
[53] ^4^	120	Male	Cobb500	Corn SBM	0–35	MHA-FA	0.502	0.315	0.486
[53] ^4^	120	Male	Cobb500	Corn SBM	0–35	MHA-FA	0.502	0.315	0.486
[53] ^4^	120	Male	Cobb500	Corn SBM	0–35	MHA-FA	0.502	0.315	0.486
[53] ^4^	120	Male	Cobb500	Corn SBM	0–35	MHA-FA	0.502	0.315	0.486
[53] ^4^	120	Male	Cobb500	Corn SBM	0–35	MHA-FA	0.502	0.315	0.486
[53] ^4^	120	Male	Cobb500	Corn SBM	0–35	MHA-FA	0.502	0.440	0.676
[53] ^4^	120	Male	Cobb500	Corn SBM	0–35	MHA-FA	0.502	0.440	0.676
[53] ^4^	120	Male	Cobb500	Corn SBM	0–35	MHA-FA	0.502	0.440	0.676
[53] ^4^	120	Male	Cobb500	Corn SBM	0–35	MHA-FA	0.502	0.440	0.676
[53] ^4^	120	Male	Cobb500	Corn SBM	0–35	MHA-FA	0.502	0.440	0.676
[53] ^4^	120	Male	Cobb500	Corn SBM	0–35	MHA-FA	0.502	0.440	0.676
[54]	110	Mixed	Cobb500	Corn SBM	0–21	MHA-FA	0.570	0.111	0.170
[54]	110	Mixed	Cobb500	Corn SBM	0–21	MHA-FA	0.570	0.221	0.340
[54]	110	Mixed	Cobb500	Corn SBM	0–21	MHA-FA	0.570	0.332	0.511
[55]	448	Male	Cobb500	Corn SBM	0–42	MHA-Ca	0.575	0.110	0.165
[55]	448	Male	Cobb500	Corn SBM	0–42	MHA-Ca	0.575	0.210	0.320
[55]	448	Male	Cobb500	Corn SBM	0–42	MHA-Ca	0.575	0.320	0.485
[56] ^4^	75	Male	Cobb500	Corn SBM	0–28	MHA-FA	0.503	0.051	0.078
[56] ^4^	75	Male	Cobb500	Corn SBM	0–28	MHA-FA	0.503	0.101	0.156
[56] ^4^	75	Male	Cobb500	Corn SBM	0–28	MHA-FA	0.503	0.169	0.260
[56] ^4^	75	Male	Cobb500	Corn SBM	0–28	MHA-FA	0.503	0.287	0.441
[57]	90	Male	Ross308	Wheat Corn SBM	0–35	MHA-FA	0.544	0.142	0.219
[57]	90	Male	Ross308	Wheat Corn SBM	0–35	MHA-FA	0.544	0.285	0.437
[57]	90	Male	Ross308	Wheat Corn SBM	0–35	MHA-FA	0.544	0.427	0.656
[58]	144	Male	Ross308	Corn SBM	0–35	MHA-FA	0.513	0.096	0.147
[58]	144	Male	Ross308	Corn SBM	0–35	MHA-FA	0.513	0.239	0.367
[58]	144	Male	Ross308	Corn SBM	0–35	MHA-FA	0.513	0.096	0.147
[58]	144	Male	Ross308	Corn SBM	0–35	MHA-FA	0.513	0.239	0.367

^1^ Without the diluent (glucose, corn starch, CaCO_3_, etc.). ^2^ Without silica. ^3^ Supplementation or dietary Met+Cys in basal diet recalculated according to AMINOChick (2012) recommendation. ^4^ SID values calculated from total AA. ^5^ SBM = soybean meal.

**Table 2:** Publication bias analysis.

Meta-Analysis	Begg and Mazumdar’s Rank Correlation Test				Egger Regression					
	Weight Gain Data		Feed Conversion Ratio Data		Weight Gain Data			Feed Conversion Ratio Data		
	Kendall’s Tau	*p*-Value	Kendall’s Tau	*p*-Value	Inter-cept	Lower to Upper CI Limit	*p*-Value	Inter-cept	Lower to Upper CI Limit	*p*-Value
Dietary Met+Cys levels	0.03	0.362	0.11	0.062	0.02	−1.40 to 1.45	0.973	0.86	−0.66 to 2.38	0.264
MHA-products	0.02	0.383	0.10	0.082	0.01	−1.43 to 1.45	0.987	0.76	−0.73 to 2.25	0.314
Publication type	0.02	0.383	0.10	0.082	0.01	−1.43 to 1.45	0.987	0.76	−0.73 to 2.25	0.314
Diet type	0.09	0.123	0.06	0.209	0.31	−1.23 to 1.85	0.686	0.50	−1.08 to 2.09	0.529
Broiler strains	0.10	0.117	0.02	0.412	0.91	−1.25 to 3.07	0.404	−0.42	−2.44 to 1.59	0.676

**Table 3:** Evaluation of the effect size on body weight gain (BWG) and forest plot of studies with different Met+Cys levels.

	**Avg. Basal** **Met+Cys, %**	**Supplementation, %**					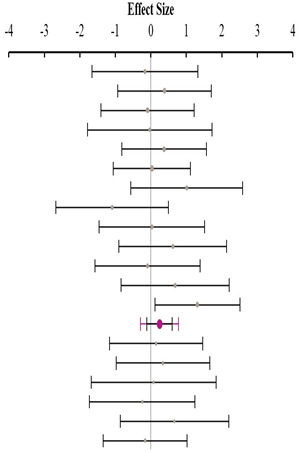
**Study Name/Subgroup Name**		**65 DLM ^1^**	**MHA ^2^**	**Effect Size**	**CI LL**	**CI UL**	
[37] ^4^	0.648	0.220	0.340	−0.17	−1.65	1.32	
[38]	0.580	0.370	0.570	0.38	−0.94	1.70	
[38]	0.520	0.310	0.476	−0.09	−1.40	1.22	
[47]	0.540	0.279	0.429	−0.03	−1.79	1.73	
[50] ^4^	0.589	0.270	0.415	0.37	−0.82	1.56	
[51]	0.540	0.279	0.429	0.03	−1.06	1.12	
[53] ^4^	0.507	0.440	0.676	1.01	−0.56	2.59	
[53] ^4^	0.507	0.440	0.676	−1.09	−2.68	0.50	
[53] ^4^	0.507	0.440	0.676	0.03	−1.45	1.51	
[53] ^4^	0.507	0.440	0.676	0.62	−0.90	2.14	
[53] ^4^	0.507	0.440	0.676	−0.09	−1.58	1.39	
[53] ^4^	0.507	0.440	0.676	0.68	−0.84	2.21	
[57]	0.544	0.427	0.656	1.31	0.11	2.51	
**Above requirement ^3^ (I^2^ = 9.7% P_Q_ = 0.348)**				**0.25**	**−0.11**	**0.60**	
[38]	0.580	0.296	0.456	0.15	−1.16	1.46	
[38]	0.520	0.248	0.381	0.34	−0.98	1.66	
[39]	0.592	0.236	0.364	0.08	−1.68	1.84	
[40]	0.591	0.238	0.366	−0.24	−1.73	1.25	
[42]	0.566	0.255	0.393	0.67	−0.86	2.19	
[43]	0.555	0.223	0.341	−0.16	−1.34	1.02	
[45] ^4^	0.648	0.180	0.278	0.74	−0.30	1.79	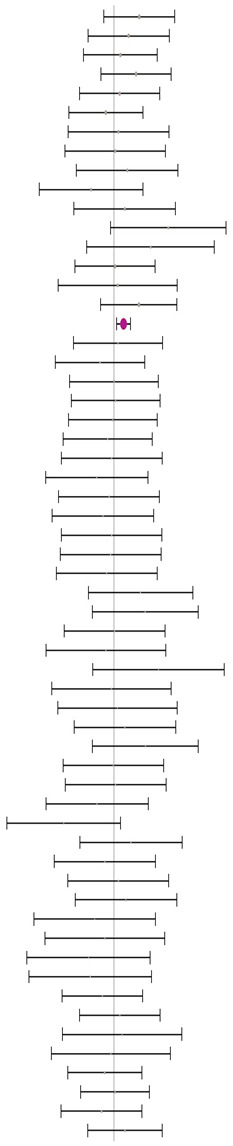
[46]	0.577	0.269	0.415	0.43	−0.77	1.63	
[48]	0.543	0.249	0.383	0.19	−0.90	1.28	
[48]	0.545	0.302	0.464	0.65	−0.39	1.68	
[50] ^4^	0.589	0.179	0.276	0.17	−1.02	1.35	
[51]	0.540	0.223	0.343	−0.24	−1.33	0.85	
[53] ^4^	0.507	0.315	0.486	0.13	−1.35	1.62	
[53] ^4^	0.507	0.315	0.486	0.03	−1.45	1.52	
[53] ^4^	0.507	0.315	0.486	0.39	−1.11	1.88	
[53] ^4^	0.507	0.315	0.486	−0.68	−2.21	0.85	
[53] ^4^	0.507	0.315	0.486	0.31	−1.18	1.81	
[53] ^4^	0.507	0.315	0.486	1.60	−0.11	3.30	
[54]	0.570	0.332	0.511	1.08	−0.80	2.96	
[55]	0.575	0.210	0.320	0.03	−1.15	1.21	
[56] ^4^	0.527	0.287	0.441	0.11	−1.65	1.86	
[57]	0.544	0.285	0.437	0.73	−0.39	1.85	
**At requirement ^3^ (I^2^ = 0.0% P_Q_ = 0.840)**				**0.28**	**0.08**	**0.48**	
[38]	0.580	0.074	0.114	0.12	−1.19	1.43	
[38]	0.580	0.148	0.228	−0.41	−1.74	0.91	
[38]	0.580	0.222	0.342	0.00	−1.31	1.31	
[38]	0.520	0.062	0.095	0.05	−1.26	1.35	
[38]	0.520	0.124	0.190	−0.03	−1.34	1.28	
[38]	0.520	0.186	0.286	−0.19	−1.50	1.13	
[35]	0.568	0.020	0.030	−0.07	−1.55	1.41	
[35]	0.568	0.039	0.060	−0.51	−2.02	1.00	
[35]	0.568	0.065	0.100	−0.15	−1.64	1.34	
[35]	0.568	0.098	0.150	−0.33	−1.82	1.17	
[35]	0.568	0.137	0.210	−0.07	−1.56	1.41	
[19]	0.555	0.026	0.040	−0.10	−1.58	1.39	
[19]	0.555	0.052	0.080	−0.21	−1.70	1.27	
[19]	0.555	0.078	0.120	0.78	−0.76	2.32	
[19]	0.555	0.104	0.160	0.92	−0.64	2.48	
[19]	0.555	0.130	0.200	0.02	−1.47	1.50	
[19]	0.505	0.039	0.060	−0.24	−2.00	1.53	
[19]	0.505	0.078	0.120	1.31	−0.63	3.25	
[19]	0.505	0.117	0.180	−0.08	−1.83	1.68	
[19]	0.505	0.156	0.240	0.10	−1.66	1.86	
[19] ^4^	0.539	0.020	0.030	0.32	−1.17	1.82	
[19] ^4^	0.539	0.039	0.060	0.92	−0.64	2.48	
[19] ^4^	0.539	0.059	0.090	−0.02	−1.50	1.47	
[19] ^4^	0.539	0.078	0.120	0.05	−1.44	1.53	
[19] ^4^	0.539	0.098	0.150	−0.50	−2.00	1.01	
[17]	0.559	0.026	0.040	−1.48	−3.16	0.19	
[17]	0.559	0.052	0.080	0.50	−1.01	2.01	
[17]	0.559	0.078	0.120	−0.28	−1.77	1.22	
[17]	0.559	0.137	0.210	0.13	−1.36	1.61	
[17]	0.559	0.195	0.300	0.35	−1.15	1.85	
[44] ^4^	0.544	0.039	0.060	−0.57	−2.36	1.22	
[44] ^4^	0.544	0.078	0.120	−0.27	−2.03	1.49	
[44] ^4^	0.544	0.117	0.180	−0.75	−2.57	1.07	
[44] ^4^	0.544	0.156	0.240	−0.70	−2.51	1.11	
[46]	0.577	0.092	0.136	−0.34	−1.53	0.85	
[46]	0.577	0.176	0.279	0.17	−1.01	1.36	
[47]	0.540	0.093	0.143	0.24	−1.52	2.00	
[47]	0.540	0.186	0.286	−0.09	−1.85	1.66	
[48]	0.543	0.086	0.133	−0.27	−1.37	0.82	
[48]	0.545	0.085	0.131	0.03	−0.99	1.04	
[50] ^4^	0.589	0.091	0.139	−0.37	−1.56	0.82	
[51]	0.540	0.112	0.172	0.32	−0.77	1.42	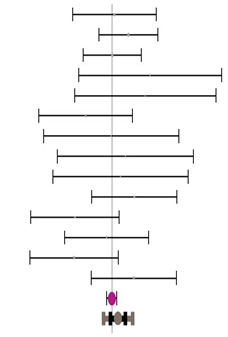
[51]	0.540	0.167	0.258	0.06	−1.02	1.15	
[52]	0.548	0.141	0.218	0.42	−0.34	1.18	
[52]	0.500	0.179	0.275	0.00	−0.75	0.75	
[54]	0.570	0.111	0.170	0.99	−0.87	2.85	
[54]	0.570	0.221	0.340	0.86	−0.98	2.70	
[55]	0.575	0.110	0.165	−0.69	−1.91	0.52	
[56] ^4^	0.527	0.051	0.078	−0.02	−1.78	1.73	
[56] ^4^	0.527	0.101	0.156	0.34	−1.43	2.11	
[56] ^4^	0.527	0.169	0.260	0.22	−1.55	1.98	
[57]	0.544	0.142	0.219	0.58	−0.53	1.68	
[58]	0.513	0.096	0.147	−0.97	−2.12	0.18	
[58]	0.513	0.239	0.367	−0.15	−1.23	0.94	
[58]	0.513	0.096	0.147	−0.99	−2.14	0.16	
[58]	0.513	0.239	0.367	0.56	−0.54	1.67	
**Below requirement ^3^ (I^2^ = 0.0% P_Q_ = 0.873)**				**−0.01**	**−0.14**	**0.12**	
**Combined effect size (I^2^ = 0.0% P_Q_ = 0.835)**				**0.15**	**−0.04**	**0.35**	

^1^ Without the diluent (glucose, corn starch, CaCO_3_, etc.). ^2^ Without silica. ^3^ Supplementation or dietary Met+Cys in basal diet recalculated according to AMINOChick^®^ 3.0 software. ^4^ SID values calculated from total AA.

**Table 4:** Evaluation of the effect size on feed conversion ratio (FCR) and forest plot of studies with different Met+Cys levels.

**Study Name/Subgroup Name**	**Avg. Basal** **Met+Cys, %**	**Supplementation, %**					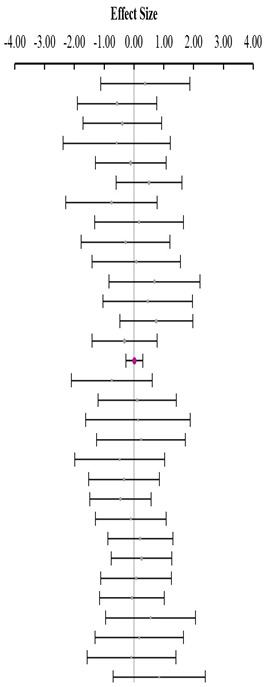
		**65 DLM ^1^**	**MHA ^2^**	**Effect Size**	**CI LL**	**CI UL**	
[37] ^4^	0.648	0.220	0.340	0.38	−1.12	1.87	
[38]	0.580	0.370	0.570	−0.57	−1.90	0.77	
[38]	0.520	0.310	0.476	−0.39	−1.71	0.93	
[47]	0.540	0.279	0.429	−0.58	−2.37	1.21	
[50] ^4^	0.589	0.270	0.415	−0.11	−1.29	1.07	
[51]	0.540	0.279	0.429	0.51	−0.60	1.61	
[53] ^4^	0.507	0.440	0.676	−0.76	−2.29	0.78	
[53] ^4^	0.507	0.440	0.676	0.17	−1.31	1.66	
[53] ^4^	0.507	0.440	0.676	−0.28	−1.77	1.21	
[53] ^4^	0.507	0.440	0.676	0.07	−1.41	1.56	
[53] ^4^	0.507	0.440	0.676	0.69	−0.84	2.22	
[53] ^4^	0.507	0.440	0.676	0.46	−1.04	1.96	
[55]	0.575	0.320	0.485	0.75	−0.47	1.97	
[57]	0.544	0.427	0.656	−0.32	−1.41	0.78	
**Above requirement ^3^ (I^2^ = 0.0% P_Q_ = 0.658)**				**0.02**	**−0.27**	**0.30**	
[38]	0.580	0.296	0.456	−0.75	−2.10	0.61	
[38]	0.520	0.248	0.381	0.11	−1.20	1.42	
[39]	0.592	0.236	0.364	0.13	−1.62	1.89	
[40]	0.591	0.238	0.366	0.24	−1.25	1.73	
[42]	0.566	0.255	0.393	−0.48	−1.98	1.03	
[43]	0.555	0.223	0.341	−0.33	−1.52	0.86	
[45] ^4^	0.648	0.180	0.278	−0.45	−1.48	0.57	
[46]	0.577	0.269	0.415	−0.11	−1.29	1.08	
[48]	0.543	0.249	0.383	0.21	−0.88	1.30	
[48]	0.545	0.302	0.464	0.25	−0.76	1.27	
[50] ^4^	0.589	0.179	0.276	0.07	−1.11	1.25	
[51]	0.540	0.223	0.343	−0.07	−1.16	1.02	
[53] ^4^	0.507	0.315	0.486	0.55	−0.96	2.07	
[53] ^4^	0.507	0.315	0.486	0.18	−1.31	1.66	
[53] ^4^	0.507	0.315	0.486	−0.08	−1.57	1.40	
[53] ^4^	0.507	0.315	0.486	0.85	−0.70	2.39	
[53] ^4^	0.507	0.315	0.486	−0.87	−2.43	0.68	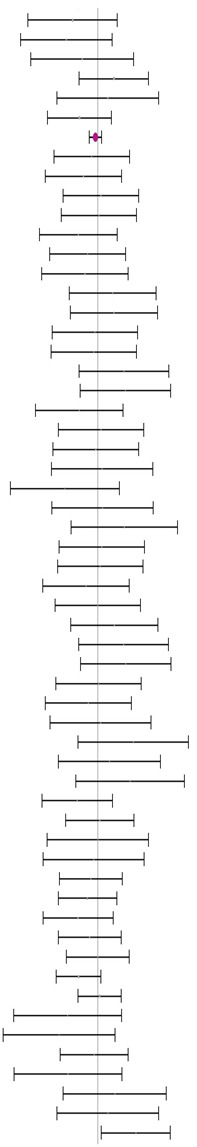
[53] ^4^	0.507	0.315	0.486	−1.09	−2.68	0.50	
[54]	0.570	0.332	0.511	−0.54	−2.33	1.25	
[55]	0.575	0.210	0.320	0.56	−0.65	1.76	
[56] ^4^	0.527	0.301	0.462	0.35	−1.42	2.12	
[57]	0.544	0.285	0.437	−0.63	−1.75	0.48	
**At requirement ^3^ (I^2^ = 0.0% P_Q_ = 0.748)**				**−0.08**	**−0.29**	**0.13**	
[38]	0.580	0.074	0.114	−0.21	−1.52	1.10	
[38]	0.580	0.148	0.228	−0.50	−1.82	0.83	
[38]	0.580	0.222	0.342	0.11	−1.20	1.42	
[38]	0.520	0.062	0.095	0.04	−1.27	1.34	
[38]	0.520	0.124	0.190	−0.67	−2.02	0.67	
[38]	0.520	0.186	0.286	−0.35	−1.67	0.96	
[35]	0.568	0.020	0.030	−0.45	−1.95	1.06	
[35]	0.568	0.039	0.060	0.51	−0.99	2.02	
[35]	0.568	0.065	0.100	0.56	−0.95	2.08	
[35]	0.568	0.098	0.150	−0.10	−1.58	1.39	
[35]	0.568	0.137	0.210	−0.14	−1.62	1.35	
[19]	0.555	0.026	0.040	0.91	−0.65	2.47	
[19]	0.555	0.052	0.080	0.96	−0.61	2.52	
[19]	0.555	0.078	0.120	−0.64	−2.17	0.88	
[19]	0.555	0.104	0.160	0.11	−1.37	1.60	
[19]	0.555	0.130	0.200	−0.07	−1.55	1.42	
[19]	0.505	0.039	0.060	0.15	−1.61	1.91	
[19]	0.505	0.078	0.120	−1.14	−3.04	0.75	
[19]	0.505	0.117	0.180	0.17	−1.59	1.92	
[19]	0.505	0.156	0.240	0.92	−0.93	2.77	
[19] ^4^	0.539	0.020	0.030	0.14	−1.35	1.63	
[19] ^4^	0.539	0.039	0.060	0.09	−1.40	1.57	
[19] ^4^	0.539	0.059	0.090	−0.41	−1.90	1.09	
[19] ^4^	0.539	0.078	0.120	0.00	−1.48	1.48	
[19] ^4^	0.539	0.098	0.150	0.57	−0.94	2.09	
[17]	0.559	0.052	0.080	0.90	−0.66	2.45	
[17]	0.559	0.078	0.120	0.97	−0.60	2.54	
[17]	0.559	0.137	0.210	0.02	−1.46	1.51	
[17]	0.559	0.195	0.300	−0.32	−1.82	1.17	
[44] ^4^	0.544	0.039	0.060	0.10	−1.66	1.85	
[44] ^4^	0.544	0.078	0.120	1.23	−0.68	3.15	
[44] ^4^	0.544	0.117	0.180	0.40	−1.37	2.18	
[44] ^4^	0.544	0.156	0.240	1.13	−0.76	3.02	
[46]	0.577	0.092	0.136	−0.71	−1.93	0.51	
[46]	0.577	0.176	0.279	0.07	−1.11	1.25	
[47]	0.540	0.093	0.143	0.00	−1.76	1.76	
[47]	0.540	0.186	0.286	−0.14	−1.90	1.61	
[48]	0.543	0.086	0.133	−0.24	−1.33	0.85	
[48]	0.545	0.085	0.131	−0.35	−1.37	0.67	
[50] ^4^	0.589	0.091	0.139	−0.68	−1.90	0.53	
[51]	0.540	0.112	0.172	−0.28	−1.37	0.81	
[51]	0.540	0.167	0.258	0.00	−1.09	1.09	
[52]	0.548	0.141	0.218	−0.66	−1.44	0.11	
[52]	0.500	0.179	0.275	0.07	−0.69	0.82	
[54]	0.570	0.111	0.170	−1.05	−2.92	0.82	
[54]	0.570	0.221	0.340	−1.34	−3.29	0.60	
[55]	0.575	0.110	0.165	−0.12	−1.31	1.06	
[56] ^4^	0.527	0.053	0.082	−1.03	−2.90	0.84	
[56] ^4^	0.527	0.107	0.164	0.59	−1.20	2.39	
[56] ^4^	0.527	0.177	0.272	0.35	−1.42	2.12	
[57]	0.544	0.142	0.219	1.32	0.12	2.52	
[58]	0.513	0.096	0.147	0.58	−0.53	1.69	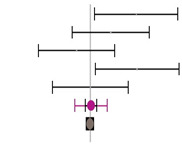
[58]	0.513	0.239	0.367	−0.40	−1.50	0.70	
[58]	0.513	0.096	0.147	1.35	0.14	2.55	
[58]	0.513	0.239	0.367	0.00	−1.09	1.09	
**Below requirement ^3^ (I^2^ = 13.3% P_Q_ = 0.205)**				**0.01**	**−0.15**	**0.17**	
**Combined effect size (I^2^ = 0.0% P_Q_ = 0.491)**				**−0.02**	**−0.08**	**0.05**	

^1^ Without the diluent (glucose, corn starch, CaCO_3_, etc.). ^2^ Without silica. ^3^ Supplementation or dietary Met+Cys in basal diet recalculated according to AMINOChick^®^ 3.0 software. ^4^ SID values calculated from total AA.

**Table 5:** Evaluation of the effect size on body weight gain (BWG) and forest plot of studies with different MHA products.

	**Avg. Basal** **Met+Cys, %**	**Supplementation, %**					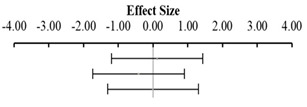
**Study Name/Subgroup Name**		**65 DLM ^1^**	**MHA ^2^**	**Effect Size**	**CI LL**	**CI UL**	
[38]	0.580	0.074	0.114	0.12	−1.19	1.43	
[38]	0.580	0.148	0.228	−0.41	−1.74	0.91	
[38]	0.580	0.222	0.342	0.00	−1.31	1.31	
[38]	0.580	0.296	0.456	0.15	−1.16	1.46	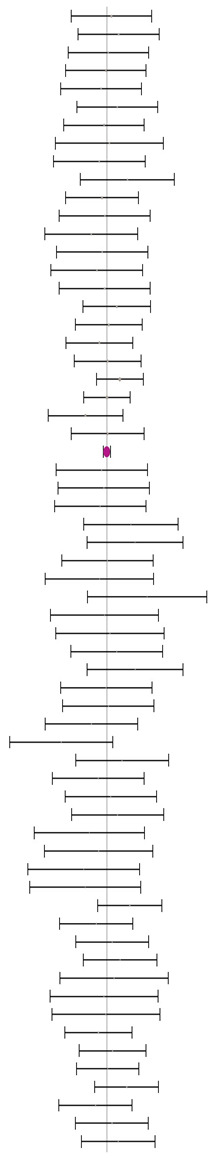
[38]	0.580	0.370	0.570	0.38	−0.94	1.70	
[38]	0.520	0.062	0.095	0.05	−1.26	1.35	
[38]	0.520	0.124	0.190	−0.03	−1.34	1.28	
[38]	0.520	0.186	0.286	−0.19	−1.50	1.13	
[38]	0.520	0.248	0.381	0.34	−0.98	1.66	
[38]	0.520	0.310	0.476	−0.09	−1.40	1.22	
[39]	0.592	0.236	0.364	0.08	−1.68	1.84	
[40] ^3^	0.591	0.238	0.366	−0.24	−1.73	1.25	
[42]	0.566	0.255	0.393	0.67	−0.86	2.19	
[43]	0.555	0.223	0.341	−0.16	−1.34	1.02	
[35]	0.568	0.020	0.030	−0.07	−1.55	1.41	
[35]	0.568	0.039	0.060	−0.51	−2.02	1.00	
[35]	0.568	0.065	0.100	−0.15	−1.64	1.34	
[35]	0.568	0.098	0.150	−0.33	−1.82	1.17	
[35]	0.568	0.137	0.210	−0.07	−1.56	1.41	
[51]	0.540	0.112	0.172	0.32	−0.77	1.42	
[51]	0.540	0.167	0.258	0.06	−1.02	1.15	
[51]	0.540	0.223	0.343	−0.24	−1.33	0.85	
[51]	0.540	0.279	0.429	0.03	−1.06	1.12	
[52]	0.548	0.141	0.218	0.42	−0.34	1.18	
[52]	0.500	0.179	0.275	0.00	−0.75	0.75	
[55]	0.575	0.110	0.165	−0.69	−1.91	0.52	
[55]	0.575	0.210	0.320	0.03	−1.15	1.21	
**MHA-Ca (I^2^ = 0.0% P_Q_ = 1.000)**				**0.00**	**−0.11**	**0.12**	
[37] ^3^	0.648	0.220	0.340	−0.17	−1.65	1.32	
[19]	0.555	0.026	0.040	−0.10	−1.58	1.39	
[19]	0.555	0.052	0.080	−0.21	−1.70	1.27	
[19]	0.555	0.078	0.120	0.78	−0.76	2.32	
[19]	0.555	0.104	0.160	0.92	−0.64	2.48	
[19]	0.555	0.130	0.200	0.02	−1.47	1.50	
[19]	0.505	0.039	0.060	−0.24	−2.00	1.53	
[19]	0.505	0.078	0.120	1.31	−0.63	3.25	
[19]	0.505	0.117	0.180	−0.08	−1.83	1.68	
[19]	0.505	0.156	0.240	0.10	−1.66	1.86	
[19] ^3^	0.539	0.020	0.030	0.32	−1.17	1.82	
[19] ^3^	0.539	0.039	0.060	0.92	−0.64	2.48	
[19] ^3^	0.539	0.059	0.090	−0.02	−1.50	1.47	
[19] ^3^	0.539	0.078	0.120	0.05	−1.44	1.53	
[19] ^3^	0.539	0.098	0.150	−0.50	−2.00	1.01	
[17]	0.559	0.026	0.040	−1.48	−3.16	0.19	
[17]	0.559	0.052	0.080	0.50	−1.01	2.01	
[17]	0.559	0.078	0.120	−0.28	−1.77	1.22	
[17]	0.559	0.137	0.210	0.13	−1.36	1.61	
[17]	0.559	0.195	0.300	0.35	−1.15	1.85	
[44] ^3^	0.544	0.039	0.060	−0.57	−2.36	1.22	
[44] ^3^	0.544	0.078	0.120	−0.27	−2.03	1.49	
[44] ^3^	0.544	0.117	0.180	−0.75	−2.57	1.07	
[44] ^3^	0.544	0.156	0.240	−0.70	−2.51	1.11	
[45] ^3^	0.648	0.180	0.278	0.74	−0.30	1.79	
[46]	0.577	0.092	0.136	−0.34	−1.53	0.85	
[46]	0.577	0.176	0.279	0.17	−1.01	1.36	
[46]	0.577	0.269	0.415	0.43	−0.77	1.63	
[47]	0.540	0.093	0.143	0.24	−1.52	2.00	
[47]	0.540	0.186	0.286	−0.09	−1.85	1.66	
[47]	0.540	0.279	0.429	−0.03	−1.79	1.73	
[48]	0.543	0.086	0.133	−0.27	−1.37	0.82	
[48]	0.543	0.249	0.383	0.19	−0.90	1.28	
[48]	0.545	0.085	0.131	0.03	−0.99	1.04	
[48]	0.545	0.302	0.464	0.65	−0.39	1.68	
[50] ^3^	0.589	0.091	0.139	−0.37	−1.56	0.82	
[50] ^3^	0.589	0.179	0.276	0.17	−1.02	1.35	
[50] ^3^	0.589	0.270	0.415	0.37	−0.82	1.56	
[53] ^3^	0.507	0.315	0.486	0.13	−1.35	1.62	
[53] ^3^	0.507	0.315	0.486	0.03	−1.45	1.52	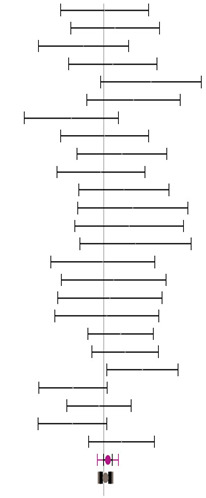
[53] ^3^	0.507	0.315	0.486	0.39	−1.11	1.88	
[53] ^3^	0.507	0.315	0.486	−0.68	−2.21	0.85	
[53] ^3^	0.507	0.315	0.486	0.31	−1.18	1.81	
[53] ^3^	0.507	0.315	0.486	1.60	−0.11	3.30	
[53] ^3^	0.507	0.440	0.676	1.01	−0.56	2.59	
[53] ^3^	0.507	0.440	0.676	−1.09	−2.68	0.50	
[53] ^3^	0.507	0.440	0.676	0.03	−1.45	1.51	
[53] ^3^	0.507	0.440	0.676	0.62	−0.90	2.14	
[53] ^3^	0.507	0.440	0.676	−0.09	−1.58	1.39	
[53] ^3^	0.507	0.440	0.676	0.68	−0.84	2.21	
[54]	0.570	0.111	0.170	0.99	−0.87	2.85	
[54]	0.570	0.221	0.340	0.86	−0.98	2.70	
[54]	0.570	0.332	0.511	1.08	−0.80	2.96	
[56] ^3^	0.527	0.053	0.082	−0.02	−1.78	1.73	
[56] ^3^	0.527	0.107	0.164	0.34	−1.43	2.11	
[56] ^3^	0.527	0.177	0.272	0.22	−1.55	1.98	
[56] ^3^	0.527	0.301	0.462	0.11	−1.65	1.86	
[57]	0.544	0.142	0.219	0.58	−0.53	1.68	
[57]	0.544	0.285	0.437	0.73	−0.39	1.85	
[57]	0.544	0.427	0.656	1.31	0.11	2.51	
[58]	0.513	0.096	0.147	−1.04	−2.19	0.12	
[58]	0.513	0.239	0.367	−0.16	−1.24	0.93	
[58]	0.513	0.096	0.147	−1.06	−2.22	0.10	
[58]	0.513	0.239	0.367	0.60	−0.51	1.71	
**MHA-FA (I^2^ = 7.7% P_Q_ = 0.303)**				**0.14**	**0.00**	**0.29**	
**Combined effect size (I^2^ = 0.0% P_Q_ = 0.809)**				**0.06**	**−0.08**	**0.21**	

^1^ Without the diluent (glucose, corn starch, CaCO_3_, etc.). ^2^ Without silica. ^3^ SID values calculated from total AA.

**Table 6:** Evaluation of the effect size on feed conversion ratio (FCR) and forest plot of studies with different MHA products.

	**Avg. Basal** **Met+Cys, %**	**Supplementation, %**					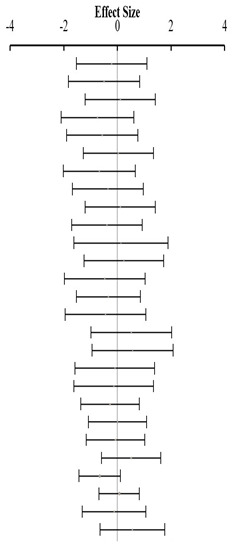
**Study Name/Subgroup Name**		**65 DLM ^1^**	**MHA ^2^**	**Effect Size**	**CI LL**	**CI UL**	
[38]	0.580	0.074	0.114	−0.21	−1.52	1.10	
[38]	0.580	0.148	0.228	−0.50	−1.82	0.83	
[38]	0.580	0.222	0.342	0.11	−1.20	1.42	
[38]	0.580	0.296	0.456	−0.75	−2.10	0.61	
[38]	0.580	0.370	0.570	−0.57	−1.90	0.77	
[38]	0.520	0.062	0.095	0.04	−1.27	1.34	
[38]	0.520	0.124	0.190	−0.67	−2.02	0.67	
[38]	0.520	0.186	0.286	−0.35	−1.67	0.96	
[38]	0.520	0.248	0.381	0.11	−1.20	1.42	
[38]	0.520	0.310	0.476	−0.39	−1.71	0.93	
[39]	0.592	0.236	0.364	0.13	−1.62	1.89	
[40] ^3^	0.591	0.238	0.366	0.24	−1.25	1.73	
[42]	0.566	0.255	0.393	−0.48	−1.98	1.03	
[43]	0.555	0.223	0.341	−0.33	−1.52	0.86	
[35]	0.568	0.020	0.030	−0.45	−1.95	1.06	
[35]	0.568	0.039	0.060	0.51	−0.99	2.02	
[35]	0.568	0.065	0.100	0.56	−0.95	2.08	
[35]	0.568	0.098	0.150	−0.10	−1.58	1.39	
[35]	0.568	0.137	0.210	−0.14	−1.62	1.35	
[51]	0.540	0.112	0.172	−0.28	−1.37	0.81	
[51]	0.540	0.167	0.258	0.00	−1.09	1.09	
[51]	0.540	0.223	0.343	−0.07	−1.16	1.02	
[51]	0.540	0.279	0.429	0.51	−0.60	1.61	
[52]	0.548	0.141	0.218	−0.66	−1.44	0.11	
[52]	0.500	0.179	0.275	0.07	−0.69	0.82	
[55]	0.575	0.110	0.165	−0.12	−1.31	1.06	
[55]	0.575	0.210	0.320	0.56	−0.65	1.76	
[55]	0.575	0.320	0.485	0.75	−0.47	1.97	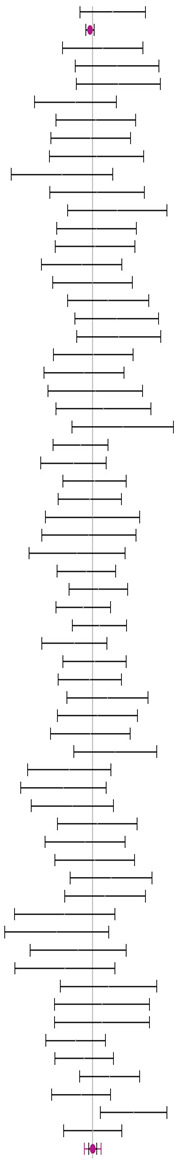
**MHA-Ca (I^2^ = 0.0% P_Q_ = 0.924)**				**−0.10**	**−0.26**	**0.06**	
[37] ^4^	0.648	0.220	0.340	0.38	−1.12	1.87	
[19]	0.555	0.026	0.040	0.91	−0.65	2.47	
[19]	0.555	0.052	0.080	0.96	−0.61	2.52	
[19]	0.555	0.078	0.120	−0.64	−2.17	0.88	
[19]	0.555	0.104	0.160	0.11	−1.37	1.60	
[19]	0.555	0.130	0.200	−0.07	−1.55	1.42	
[19]	0.505	0.039	0.060	0.15	−1.61	1.91	
[19]	0.505	0.078	0.120	−1.14	−3.04	0.75	
[19]	0.505	0.117	0.180	0.17	−1.59	1.92	
[19]	0.505	0.156	0.240	0.92	−0.93	2.77	
[19] ^4^	0.539	0.020	0.030	0.14	−1.35	1.63	
[19] ^4^	0.539	0.039	0.060	0.09	−1.40	1.57	
[19] ^4^	0.539	0.059	0.090	−0.41	−1.90	1.09	
[19] ^4^	0.539	0.078	0.120	0.00	−1.48	1.48	
[19] ^4^	0.539	0.098	0.150	0.57	−0.94	2.09	
[17]	0.559	0.052	0.080	0.90	−0.66	2.45	
[17]	0.559	0.078	0.120	0.97	−0.60	2.54	
[17]	0.559	0.137	0.210	0.02	−1.46	1.51	
[17]	0.559	0.195	0.300	−0.32	−1.82	1.17	
[44] ^4^	0.544	0.039	0.060	0.10	−1.66	1.85	
[44] ^4^	0.544	0.117	0.180	0.40	−1.37	2.18	
[44] ^4^	0.544	0.156	0.240	1.13	−0.76	3.02	
[45] ^4^	0.648	0.180	0.278	−0.45	−1.48	0.57	
[46]	0.577	0.092	0.136	−0.71	−1.93	0.51	
[46]	0.577	0.176	0.279	0.07	−1.11	1.25	
[46]	0.577	0.269	0.415	−0.11	−1.29	1.08	
[47]	0.540	0.093	0.143	0.00	−1.76	1.76	
[47]	0.540	0.186	0.286	−0.14	−1.90	1.61	
[47]	0.540	0.279	0.429	−0.58	−2.37	1.21	
[48]	0.543	0.086	0.133	−0.24	−1.33	0.85	
[48]	0.543	0.249	0.383	0.21	−0.88	1.30	
[48]	0.545	0.085	0.131	−0.35	−1.37	0.67	
[48]	0.545	0.302	0.464	0.25	−0.76	1.27	
[50] ^4^	0.589	0.091	0.139	−0.68	−1.90	0.53	
[50] ^4^	0.589	0.179	0.276	0.07	−1.11	1.25	
[50] ^4^	0.589	0.270	0.415	−0.11	−1.29	1.07	
[53] ^4^	0.507	0.315	0.486	0.55	−0.96	2.07	
[53] ^4^	0.507	0.315	0.486	0.18	−1.31	1.66	
[53] ^4^	0.507	0.315	0.486	−0.08	−1.57	1.40	
[53] ^4^	0.507	0.315	0.486	0.85	−0.70	2.39	
[53] ^4^	0.507	0.315	0.486	−0.87	−2.43	0.68	
[53] ^4^	0.507	0.315	0.486	−1.09	−2.68	0.50	
[53] ^4^	0.507	0.440	0.676	−0.76	−2.29	0.78	
[53] ^4^	0.507	0.440	0.676	0.17	−1.31	1.66	
[53] ^4^	0.507	0.440	0.676	−0.28	−1.77	1.21	
[53] ^4^	0.507	0.440	0.676	0.07	−1.41	1.56	
[53] ^4^	0.507	0.440	0.676	0.69	−0.84	2.22	
[53] ^4^	0.507	0.440	0.676	0.46	−1.04	1.96	
[54]	0.570	0.111	0.170	−1.05	−2.92	0.82	
[54]	0.570	0.221	0.340	−1.34	−3.29	0.60	
[54]	0.570	0.332	0.511	−0.54	−2.33	1.25	
[56] ^4^	0.527	0.053	0.082	−1.03	−2.90	0.84	
[56] ^4^	0.527	0.107	0.164	0.59	−1.20	2.39	
[56] ^4^	0.527	0.177	0.272	0.35	−1.42	2.12	
[56] ^4^	0.527	0.301	0.462	0.35	−1.42	2.12	
[57]	0.544	0.285	0.437	−0.63	−1.75	0.48	
[57]	0.544	0.427	0.656	−0.32	−1.41	0.78	
[58]	0.513	0.096	0.147	0.63	−0.48	1.75	
[58]	0.513	0.239	0.367	−0.43	−1.53	0.67	
[58]	0.513	0.096	0.147	1.53	0.29	2.77	
[58]	0.513	0.239	0.367	0.00	−1.09	1.09	
**MHA-FA (I^2^ = 5.5% P_Q_ = 0.354)**				**0.00**	**−0.15**	**0.15**	
**Combined effect size (I^2^ = 0.0% P_Q_ = 0.674)**				**−0.05**	**−0.15**	**0.05**	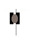

^1^ Without the diluent (glucose, corn starch, CaCO_3_, etc.). ^2^ Without silica. ^3^ Supplementation or dietary Met+Cys in basal diet recalculated according to AMINOChick^®^ 3.0 software. ^4^ SID values calculated from total AA.

**Table 7:** Evaluation of the effect size on body weight gain (BWG) and forest plot of studies from in-house data and peer-reviewed papers.

	**Avg. Basal** **Met+Cys, %**	**Supplementation, %**					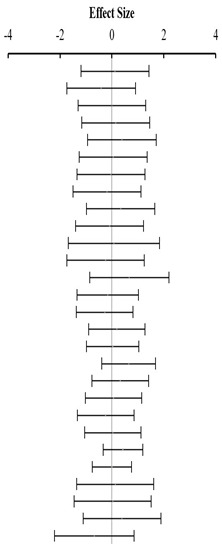
**Study Name/Subgroup Name**		**65 DLM ^1^**	**MHA ^2^**	**Effect Size**	**CI LL**	**CI UL**	
[38]	0.580	0.074	0.114	0.12	−1.19	1.43	
[38]	0.580	0.148	0.228	−0.41	−1.74	0.91	
[38]	0.580	0.222	0.342	0.00	−1.31	1.31	
[38]	0.580	0.296	0.456	0.15	−1.16	1.46	
[38]	0.580	0.370	0.570	0.38	−0.94	1.70	
[38]	0.520	0.062	0.095	0.05	−1.26	1.35	
[38]	0.520	0.124	0.190	−0.03	−1.34	1.28	
[38]	0.520	0.186	0.286	−0.19	−1.50	1.13	
[38]	0.520	0.248	0.381	0.34	−0.98	1.66	
[38]	0.520	0.310	0.476	−0.09	−1.40	1.22	
[39]	0.592	0.236	0.364	0.08	−1.68	1.84	
[40] ^3^	0.591	0.238	0.366	−0.24	−1.73	1.25	
[42]	0.566	0.255	0.393	0.67	−0.86	2.19	
[43]	0.555	0.223	0.341	−0.16	−1.34	1.02	
[48]	0.543	0.086	0.133	−0.27	−1.37	0.82	
[48]	0.543	0.249	0.383	0.19	−0.90	1.28	
[48]	0.545	0.085	0.131	0.03	−0.99	1.04	
[48]	0.545	0.302	0.464	0.65	−0.39	1.68	
[51]	0.540	0.112	0.172	0.32	−0.77	1.42	
[51]	0.540	0.167	0.258	0.06	−1.02	1.15	
[51]	0.540	0.223	0.343	−0.24	−1.33	0.85	
[51]	0.540	0.279	0.429	0.03	−1.06	1.12	
[52]	0.548	0.141	0.218	0.42	−0.34	1.18	
[52]	0.500	0.179	0.275	0.00	−0.75	0.75	
[53] ^4^	0.507	0.315	0.486	0.13	−1.35	1.62	
[53] ^4^	0.507	0.315	0.486	0.03	−1.45	1.52	
[53] ^4^	0.507	0.315	0.486	0.39	−1.11	1.88	
[53] ^4^	0.507	0.315	0.486	−0.68	−2.21	0.85	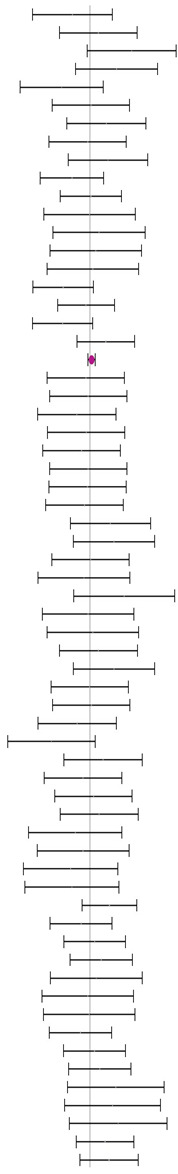
[53] ^4^	0.507	0.315	0.486	0.31	−1.18	1.81	
[53] ^4^	0.507	0.315	0.486	1.60	−0.11	3.30	
[53] ^4^	0.507	0.440	0.676	1.01	−0.56	2.59	
[53] ^4^	0.507	0.440	0.676	−1.09	−2.68	0.50	
[53] ^4^	0.507	0.440	0.676	0.03	−1.45	1.51	
[53] ^4^	0.507	0.440	0.676	0.62	−0.90	2.14	
[53] ^4^	0.507	0.440	0.676	−0.09	−1.58	1.39	
[53] ^4^	0.507	0.440	0.676	0.68	−0.84	2.21	
[55]	0.575	0.110	0.165	−0.69	−1.91	0.52	
[55]	0.575	0.210	0.320	0.03	−1.15	1.21	
[56] ^4^	0.527	0.053	0.082	−0.02	−1.78	1.73	
[56] ^4^	0.527	0.107	0.164	0.34	−1.43	2.11	
[56] ^4^	0.527	0.177	0.272	0.22	−1.55	1.98	
[56] ^4^	0.527	0.301	0.462	0.11	−1.65	1.86	
[58]	0.513	0.096	0.147	−1.04	−2.19	0.12	
[58]	0.513	0.239	0.367	−0.16	−1.24	0.93	
[58]	0.513	0.096	0.147	−1.06	−2.22	0.10	
[58]	0.513	0.239	0.367	0.60	−0.51	1.71	
**In-house (I^2^ = 0.0% P_Q_ = 0.821)**				**0.05**	**−0.09**	**0.19**	
[37] ^4^	0.648	0.220	0.340	−0.17	−1.65	1.32	
[35]	0.568	0.020	0.030	−0.07	−1.55	1.41	
[35]	0.568	0.039	0.060	−0.51	−2.02	1.00	
[35]	0.568	0.065	0.100	−0.15	−1.64	1.34	
[35]	0.568	0.098	0.150	−0.33	−1.82	1.17	
[35]	0.568	0.137	0.210	−0.07	−1.56	1.41	
[19]	0.555	0.026	0.040	−0.10	−1.58	1.39	
[19]	0.555	0.052	0.080	−0.21	−1.70	1.27	
[19]	0.555	0.078	0.120	0.78	−0.76	2.32	
[19]	0.555	0.104	0.160	0.92	−0.64	2.48	
[19]	0.555	0.130	0.200	0.02	−1.47	1.50	
[19]	0.505	0.039	0.060	−0.24	−2.00	1.53	
[19]	0.505	0.078	0.120	1.31	−0.63	3.25	
[19]	0.505	0.117	0.180	−0.08	−1.83	1.68	
[19]	0.505	0.156	0.240	0.10	−1.66	1.86	
[19] ^4^	0.539	0.020	0.030	0.32	−1.17	1.82	
[19] ^4^	0.539	0.039	0.060	0.92	−0.64	2.48	
[19] ^4^	0.539	0.059	0.090	−0.02	−1.50	1.47	
[19] ^4^	0.539	0.078	0.120	0.05	−1.44	1.53	
[19] ^4^	0.539	0.098	0.150	−0.50	−2.00	1.01	
[17]	0.559	0.026	0.040	−1.48	−3.16	0.19	
[17]	0.559	0.052	0.080	0.50	−1.01	2.01	
[17]	0.559	0.078	0.120	−0.28	−1.77	1.22	
[17]	0.559	0.137	0.210	0.13	−1.36	1.61	
[17]	0.559	0.195	0.300	0.35	−1.15	1.85	
[44] ^4^	0.544	0.039	0.060	−0.57	−2.36	1.22	
[44] ^4^	0.544	0.078	0.120	−0.27	−2.03	1.49	
[44] ^4^	0.544	0.117	0.180	−0.75	−2.57	1.07	
[44] ^4^	0.544	0.156	0.240	−0.70	−2.51	1.11	
[45] ^4^	0.648	0.180	0.278	0.74	−0.30	1.79	
[46]	0.577	0.092	0.136	−0.34	−1.53	0.85	
[46]	0.577	0.176	0.279	0.17	−1.01	1.36	
[46]	0.577	0.269	0.415	0.43	−0.77	1.63	
[47]	0.54	0.093	0.143	0.24	−1.52	2.00	
[47]	0.54	0.186	0.286	−0.09	−1.85	1.66	
[47]	0.54	0.279	0.429	−0.03	−1.79	1.73	
[50] ^4^	0.589	0.091	0.139	−0.37	−1.56	0.82	
[50] ^4^	0.589	0.179	0.276	0.17	−1.02	1.35	
[50] ^4^	0.589	0.270	0.415	0.37	−0.82	1.56	
[54]	0.570	0.111	0.170	0.99	−0.87	2.85	
[54]	0.570	0.221	0.340	0.86	−0.98	2.70	
[54]	0.570	0.332	0.511	1.08	−0.80	2.96	
[57]	0.544	0.142	0.219	0.58	−0.53	1.68	
[57]	0.544	0.285	0.437	0.73	−0.39	1.85	
[57]	0.544	0.427	0.656	1.31	0.11	2.51	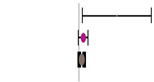
**Peer-reviewed Paper (I^2^ = 0.0% P_Q_ = 0.590)**				**0.15**	**−0.02**	**0.31**	
**Combined effect size (I^2^ = 0.0% P_Q_ = 0.809)**				**0.09**	**0.00**	**0.18**	

^1^ Without the diluent (glucose, corn starch, CaCO_3_, etc.). ^2^ Without silica. ^3^ Supplementation or dietary Met+Cys in basal diet recalculated according to AMINOChick^®^ 3.0 software. ^4^ SID values calculated from total AA.

**Table 8:** Evaluation of the effect size on feed conversion ratio (FCR) and forest plot of studies from in-house data and peer-reviewed papers.

	**Avg. Basal** **Met+Cys, %**	**Supplementation, %**					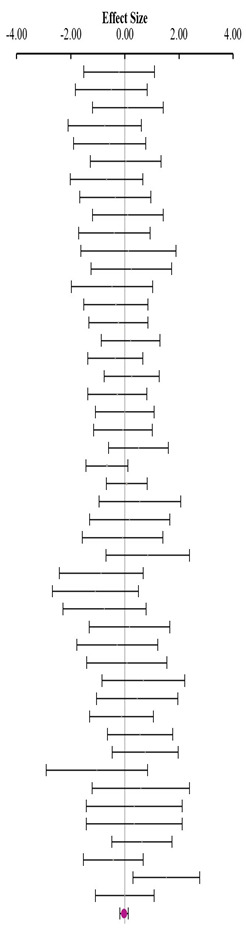
**Study Name/Subgroup Name**		**65 DLM ^1^**	**MHA ^2^**	**Effect Size**	**CI LL**	**CI UL**	
[38]	0.580	0.074	0.114	−0.21	−1.52	1.10	
[38]	0.580	0.148	0.228	−0.50	−1.82	0.83	
[38]	0.580	0.222	0.342	0.11	−1.20	1.42	
[38]	0.580	0.296	0.456	−0.75	−2.10	0.61	
[38]	0.580	0.370	0.570	−0.57	−1.90	0.77	
[38]	0.520	0.062	0.095	0.04	−1.27	1.34	
[38]	0.520	0.124	0.190	−0.67	−2.02	0.67	
[38]	0.520	0.186	0.286	−0.35	−1.67	0.96	
[38]	0.520	0.248	0.381	0.11	−1.20	1.42	
[38]	0.520	0.310	0.476	−0.39	−1.71	0.93	
[39]	0.592	0.236	0.364	0.13	−1.62	1.89	
[40] ^3^	0.591	0.238	0.366	0.24	−1.25	1.73	
[42]	0.566	0.255	0.393	−0.48	−1.98	1.03	
[43]	0.555	0.223	0.341	−0.33	−1.52	0.86	
[48]	0.543	0.086	0.133	−0.24	−1.33	0.85	
[48]	0.543	0.249	0.383	0.21	−0.88	1.30	
[48]	0.545	0.085	0.131	−0.35	−1.37	0.67	
[48]	0.545	0.302	0.464	0.25	−0.76	1.27	
[51]	0.540	0.112	0.172	−0.28	−1.37	0.81	
[51]	0.540	0.167	0.258	0.00	−1.09	1.09	
[51]	0.540	0.223	0.343	−0.07	−1.16	1.02	
[51]	0.540	0.279	0.429	0.51	−0.60	1.61	
[52]	0.548	0.141	0.218	−0.66	−1.44	0.11	
[52]	0.500	0.179	0.275	0.07	−0.69	0.82	
[53] ^4^	0.507	0.315	0.486	0.55	−0.96	2.07	
[53] ^4^	0.507	0.315	0.486	0.18	−1.31	1.66	
[53] ^4^	0.507	0.315	0.486	−0.08	−1.57	1.40	
[53] ^4^	0.507	0.315	0.486	0.85	−0.70	2.39	
[53] ^4^	0.507	0.315	0.486	−0.87	−2.43	0.68	
[53] ^4^	0.507	0.315	0.486	−1.09	−2.68	0.50	
[53] ^4^	0.507	0.440	0.676	−0.76	−2.29	0.78	
[53] ^4^	0.507	0.440	0.676	0.17	−1.31	1.66	
[53] ^4^	0.507	0.440	0.676	−0.28	−1.77	1.21	
[53] ^4^	0.507	0.440	0.676	0.07	−1.41	1.56	
[53] ^4^	0.507	0.440	0.676	0.69	−0.84	2.22	
[53] ^4^	0.507	0.440	0.676	0.46	−1.04	1.96	
[55]	0.575	0.110	0.165	−0.12	−1.31	1.06	
[55]	0.575	0.210	0.320	0.56	−0.65	1.76	
[55]	0.575	0.320	0.485	0.75	−0.47	1.97	
[56] ^4^	0.527	0.053	0.082	−1.03	−2.90	0.84	
[56] ^4^	0.527	0.107	0.164	0.59	−1.20	2.39	
[56] ^4^	0.527	0.177	0.272	0.35	−1.42	2.12	
[56] ^4^	0.527	0.301	0.462	0.35	−1.42	2.12	
[58]	0.513	0.096	0.147	0.63	−0.48	1.75	
[58]	0.513	0.239	0.367	−0.43	−1.53	0.67	
[58]	0.513	0.096	0.147	1.53	0.29	2.77	
[58]	0.513	0.239	0.367	0.00	−1.09	1.09	
**In-house (I^2^ = 0.0% P_Q_ = 0.567)**				**−0.03**	**−0.18**	**0.12**	
[37] ^4^	0.648	0.220	0.340	0.38	−1.12	1.87	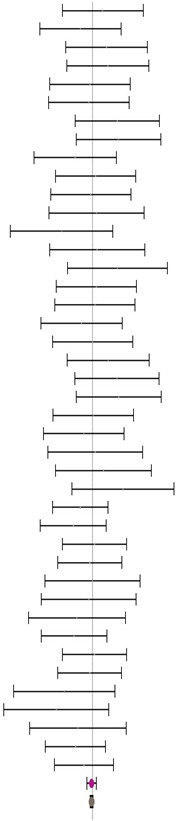
[35]	0.568	0.020	0.030	−0.45	−1.95	1.06	
[35]	0.568	0.039	0.060	0.51	−0.99	2.02	
[35]	0.568	0.065	0.100	0.56	−0.95	2.08	
[35]	0.568	0.098	0.150	−0.10	−1.58	1.39	
[35]	0.568	0.137	0.210	−0.14	−1.62	1.35	
[19]	0.555	0.026	0.040	0.91	−0.65	2.47	
[19]	0.555	0.052	0.080	0.96	−0.61	2.52	
[19]	0.555	0.078	0.120	−0.64	−2.17	0.88	
[19]	0.555	0.104	0.160	0.11	−1.37	1.60	
[19]	0.555	0.130	0.200	−0.07	−1.55	1.42	
[19]	0.505	0.039	0.060	0.15	−1.61	1.91	
[19]	0.505	0.078	0.120	−1.14	−3.04	0.75	
[19]	0.505	0.117	0.180	0.17	−1.59	1.92	
[19]	0.505	0.156	0.240	0.92	−0.93	2.77	
[19] ^4^	0.539	0.020	0.030	0.14	−1.35	1.63	
[19] ^4^	0.539	0.039	0.060	0.09	−1.40	1.57	
[19] ^4^	0.539	0.059	0.090	−0.41	−1.90	1.09	
[19] ^4^	0.539	0.078	0.120	0.00	−1.48	1.48	
[19] ^4^	0.539	0.098	0.150	0.57	−0.94	2.09	
[17]	0.559	0.052	0.080	0.90	−0.66	2.45	
[17]	0.559	0.078	0.120	0.97	−0.60	2.54	
[17]	0.559	0.137	0.210	0.02	−1.46	1.51	
[17]	0.559	0.195	0.300	−0.32	−1.82	1.17	
[44] ^4^	0.544	0.039	0.060	0.10	−1.66	1.85	
[44] ^4^	0.544	0.117	0.180	0.40	−1.37	2.18	
[44] ^4^	0.544	0.156	0.240	1.13	−0.76	3.02	
[45] ^4^	0.648	0.180	0.278	−0.45	−1.48	0.57	
[46]	0.577	0.092	0.136	−0.71	−1.93	0.51	
[46]	0.577	0.176	0.279	0.07	−1.11	1.25	
[46]	0.577	0.269	0.415	−0.11	−1.29	1.08	
[47]	0.54	0.093	0.143	0.00	−1.76	1.76	
[47]	0.54	0.186	0.286	−0.14	−1.90	1.61	
[47]	0.54	0.279	0.429	−0.58	−2.37	1.21	
[50] ^4^	0.589	0.091	0.139	−0.68	−1.90	0.53	
[50] ^4^	0.589	0.179	0.276	0.07	−1.11	1.25	
[50] ^4^	0.589	0.270	0.415	−0.11	−1.29	1.07	
[54]	0.570	0.111	0.170	−1.05	−2.92	0.82	
[54]	0.570	0.221	0.340	−1.34	−3.29	0.60	
[54]	0.570	0.332	0.511	−0.54	−2.33	1.25	
[57]	0.544	0.285	0.437	−0.63	−1.75	0.48	
[57]	0.544	0.427	0.656	−0.32	−1.41	0.78	
**Peer-reviewed Paper (I^2^ = 0.0% P_Q_ = 0.617)**				**−0.04**	**−0.21**	**0.13**	
**Combined effect size (I^2^ = 0.0% P_Q_ = 0.674)**				**−0.03**	**−0.04**	**−0.03**	

^1^ Without the diluent (glucose, corn starch, CaCO_3_, etc.). ^2^ Without silica. ^3^ Supplementation or dietary Met+Cys in basal diet recalculated according to AMINOChick^®^ 3.0 software. ^4^ SID values calculated from total AA.

**Table 9:** Evaluation of the effect size on body weight gain (BWG) and forest plot of studies with different broiler breeds.

	**Avg. Basal** **Met+Cys, %**	**Supplementation, %**					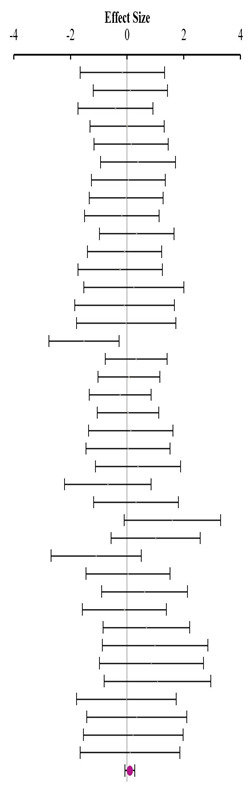
**Study Name/Subgroup Name**		**65 DLM ^1^**	**MHA ^2^**	**Effect Size**	**CI LL**	**CI UL**	
[37] ^3^	0.648	0.220	0.340	−0.17	−1.65	1.32	
[38]	0.580	0.074	0.114	0.12	−1.19	1.43	
[38]	0.580	0.148	0.228	−0.41	−1.74	0.91	
[38]	0.580	0.222	0.342	0.00	−1.31	1.31	
[38]	0.580	0.296	0.456	0.15	−1.16	1.46	
[38]	0.580	0.370	0.570	0.38	−0.94	1.70	
[38]	0.520	0.062	0.095	0.05	−1.26	1.35	
[38]	0.520	0.124	0.190	−0.03	−1.34	1.28	
[38]	0.520	0.186	0.286	−0.19	−1.50	1.13	
[38]	0.520	0.248	0.381	0.34	−0.98	1.66	
[38]	0.520	0.310	0.476	−0.09	−1.40	1.22	
[40]	0.591	0.238	0.366	−0.24	−1.73	1.25	
[47]	0.540	0.093	0.143	0.24	−1.52	2.00	
[47]	0.540	0.186	0.286	−0.09	−1.85	1.66	
[47]	0.540	0.279	0.429	−0.03	−1.79	1.73	
[51]	0.540	0.056	0.086	−1.52	−2.76	−0.29	
[51]	0.540	0.112	0.172	0.32	−0.77	1.42	
[51]	0.540	0.167	0.258	0.06	−1.02	1.15	
[51]	0.540	0.223	0.343	−0.24	−1.33	0.85	
[51]	0.540	0.279	0.429	0.03	−1.06	1.12	
[53] ^3^	0.507	0.315	0.486	0.13	−1.35	1.62	
[53] ^3^	0.507	0.315	0.486	0.03	−1.45	1.52	
[53] ^3^	0.507	0.315	0.486	0.39	−1.11	1.88	
[53] ^3^	0.507	0.315	0.486	−0.68	−2.21	0.85	
[53] ^3^	0.507	0.315	0.486	0.31	−1.18	1.81	
[53] ^3^	0.507	0.315	0.486	1.60	−0.11	3.30	
[53] ^3^	0.507	0.440	0.676	1.01	−0.56	2.59	
[53] ^3^	0.507	0.440	0.676	−1.09	−2.68	0.50	
[53] ^3^	0.507	0.440	0.676	0.03	−1.45	1.51	
[53] ^3^	0.507	0.440	0.676	0.62	−0.90	2.14	
[53] ^3^	0.507	0.440	0.676	−0.09	−1.58	1.39	
[53] ^3^	0.507	0.440	0.676	0.68	−0.84	2.21	
[54]	0.570	0.111	0.170	0.99	−0.87	2.85	
[54]	0.570	0.221	0.340	0.86	−0.98	2.70	
[54]	0.570	0.332	0.511	1.08	−0.80	2.96	
[56] ^3^	0.527	0.053	0.082	−0.02	−1.78	1.73	
[56] ^3^	0.527	0.107	0.164	0.34	−1.43	2.11	
[56] ^3^	0.527	0.177	0.272	0.22	−1.55	1.98	
[56] ^3^	0.527	0.301	0.462	0.11	−1.65	1.86	
**Cobb500 (I^2^ = 0.0% P_Q_ = 0.701)**				**0.09**	**−0.08**	**0.27**	
[39]	0.592	0.236	0.364	0.08	−1.68	1.84	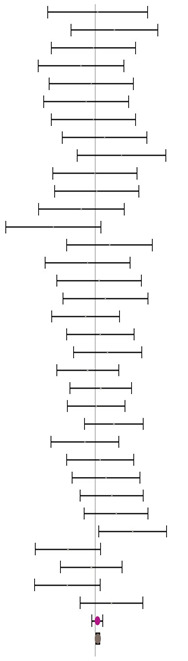
[42]	0.566	0.255	0.393	0.67	−0.86	2.19	
[35]	0.568	0.020	0.030	−0.07	−1.55	1.41	
[35]	0.568	0.039	0.060	−0.51	−2.02	1.00	
[35]	0.568	0.065	0.100	−0.15	−1.64	1.34	
[35]	0.568	0.098	0.150	−0.33	−1.82	1.17	
[35]	0.568	0.137	0.210	−0.07	−1.56	1.41	
[19] ^3^	0.539	0.020	0.030	0.32	−1.17	1.82	
[19] ^3^	0.539	0.039	0.060	0.92	−0.64	2.48	
[19] ^3^	0.539	0.059	0.090	−0.02	−1.50	1.47	
[19] ^3^	0.539	0.078	0.120	0.05	−1.44	1.53	
[19] ^3^	0.539	0.098	0.150	−0.50	−2.00	1.01	
[17]	0.559	0.026	0.040	−1.48	−3.16	0.19	
[17]	0.559	0.052	0.080	0.50	−1.01	2.01	
[17]	0.559	0.078	0.120	−0.28	−1.77	1.22	
[17]	0.559	0.137	0.210	0.13	−1.36	1.61	
[17]	0.559	0.195	0.300	0.35	−1.15	1.85	
[46]	0.577	0.092	0.136	−0.34	−1.53	0.85	
[46]	0.577	0.176	0.279	0.17	−1.01	1.36	
[46]	0.577	0.269	0.415	0.43	−0.77	1.63	
[48]	0.543	0.086	0.133	−0.27	−1.37	0.82	
[48]	0.543	0.249	0.383	0.19	−0.90	1.28	
[48]	0.545	0.085	0.131	0.03	−0.99	1.04	
[48]	0.545	0.302	0.464	0.65	−0.39	1.68	
[50] ^3^	0.589	0.091	0.139	−0.37	−1.56	0.82	
[50] ^3^	0.589	0.179	0.276	0.17	−1.02	1.35	
[50] ^3^	0.589	0.270	0.415	0.37	−0.82	1.56	
[57]	0.544	0.142	0.219	0.58	−0.53	1.68	
[57]	0.544	0.285	0.437	0.73	−0.39	1.85	
[57]	0.544	0.427	0.656	1.31	0.11	2.51	
[58]	0.513	0.096	0.147	−0.97	−2.12	0.18	
[58]	0.513	0.239	0.367	−0.15	−1.23	0.94	
[58]	0.513	0.096	0.147	−0.99	−2.14	0.16	
[58]	0.513	0.239	0.367	0.56	−0.54	1.67	
**Ross308 (I^2^ = 8.3% P_Q_ = 0.330)**				**0.07**	**−0.12**	**0.26**	
**Combined effect size (I^2^ = 0.0% P_Q_ = 0.578)**				**0.08**	**0.06**	**0.11**	

^1^ Without the diluent (glucose, corn starch, CaCO_3_, etc.). ^2^ Without silica. ^3^ SID values calculated from total AA.

**Table 10:** Evaluation of the effect size on feed conversion ratio (FCR) and forest plot of studies with different broiler breeds.

	**Avg. Basal** **Met+Cys, %**	**Supplementation, %**					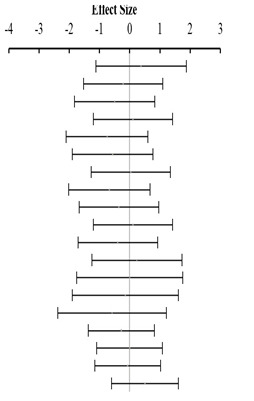
**Study Name/Subgroup Name**		**65 DLM ^1^**	**MHA ^2^**	**Effect Size**	**CI LL**	**CI UL**	
[37] ^3^	0.648	0.220	0.340	0.38	−1.12	1.87	
[38]	0.580	0.074	0.114	−0.21	−1.52	1.10	
[38]	0.580	0.148	0.228	−0.50	−1.82	0.83	
[38]	0.580	0.222	0.342	0.11	−1.20	1.42	
[38]	0.580	0.296	0.456	−0.75	−2.10	0.61	
[38]	0.580	0.370	0.570	−0.57	−1.90	0.77	
[38]	0.520	0.062	0.095	0.04	−1.27	1.34	
[38]	0.520	0.124	0.190	−0.67	−2.02	0.67	
[38]	0.520	0.186	0.286	−0.35	−1.67	0.96	
[38]	0.520	0.248	0.381	0.11	−1.20	1.42	
[38]	0.520	0.310	0.476	−0.39	−1.71	0.93	
[40]	0.591	0.238	0.366	0.24	−1.25	1.73	
[47]	0.540	0.093	0.143	0.00	−1.76	1.76	
[47]	0.540	0.186	0.286	−0.14	−1.90	1.61	
[47]	0.540	0.279	0.429	−0.58	−2.37	1.21	
[51]	0.540	0.112	0.172	−0.28	−1.37	0.81	
[51]	0.540	0.167	0.258	0.00	−1.09	1.09	
[51]	0.540	0.223	0.343	−0.07	−1.16	1.02	
[51]	0.540	0.279	0.429	0.51	−0.60	1.61	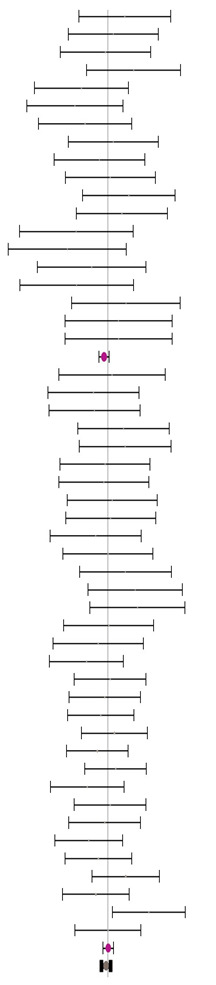
[53] ^3^	0.507	0.315	0.486	0.55	−0.96	2.07	
[53] ^3^	0.507	0.315	0.486	0.18	−1.31	1.66	
[53] ^3^	0.507	0.315	0.486	−0.08	−1.57	1.40	
[53] ^3^	0.507	0.315	0.486	0.85	−0.70	2.39	
[53] ^3^	0.507	0.315	0.486	−0.87	−2.43	0.68	
[53] ^3^	0.507	0.315	0.486	−1.09	−2.68	0.50	
[53] ^3^	0.507	0.440	0.676	−0.76	−2.29	0.78	
[53] ^3^	0.507	0.440	0.676	0.17	−1.31	1.66	
[53] ^3^	0.507	0.440	0.676	−0.28	−1.77	1.21	
[53] ^3^	0.507	0.440	0.676	0.07	−1.41	1.56	
[53] ^3^	0.507	0.440	0.676	0.69	−0.84	2.22	
[53] ^3^	0.507	0.440	0.676	0.46	−1.04	1.96	
[54]	0.570	0.111	0.170	−1.05	−2.92	0.82	
[54]	0.570	0.221	0.340	−1.34	−3.29	0.60	
[54]	0.570	0.332	0.511	−0.54	−2.33	1.25	
[56] ^3^	0.527	0.053	0.082	−1.03	−2.90	0.84	
[56] ^3^	0.527	0.107	0.164	0.59	−1.20	2.39	
[56] ^3^	0.527	0.177	0.272	0.35	−1.42	2.12	
[56] ^3^	0.527	0.301	0.462	0.35	−1.42	2.12	
**Cobb500 (I^2^ = 0.0% P_Q_ = 0.787)**				**−0.13**	**−0.30**	**0.04**	
[39]	0.592	0.236	0.364	0.13	−1.62	1.89	
[42]	0.566	0.255	0.393	−0.48	−1.98	1.03	
[35]	0.568	0.020	0.030	−0.45	−1.95	1.06	
[35]	0.568	0.039	0.060	0.51	−0.99	2.02	
[35]	0.568	0.065	0.100	0.56	−0.95	2.08	
[35]	0.568	0.098	0.150	−0.10	−1.58	1.39	
[35]	0.568	0.137	0.210	−0.14	−1.62	1.35	
[19] ^3^	0.539	0.020	0.030	0.14	−1.35	1.63	
[19] ^3^	0.539	0.039	0.060	0.09	−1.40	1.57	
[19] ^3^	0.539	0.059	0.090	−0.41	−1.90	1.09	
[19] ^3^	0.539	0.078	0.120	0.00	−1.48	1.48	
[19] ^3^	0.539	0.098	0.150	0.57	−0.94	2.09	
[17]	0.559	0.052	0.080	0.90	−0.66	2.45	
[17]	0.559	0.078	0.120	0.97	−0.60	2.54	
[17]	0.559	0.137	0.210	0.02	−1.46	1.51	
[17]	0.559	0.195	0.300	−0.32	−1.82	1.17	
[46]	0.577	0.092	0.136	−0.71	−1.93	0.51	
[46]	0.577	0.176	0.279	0.07	−1.11	1.25	
[46]	0.577	0.269	0.415	−0.11	−1.29	1.08	
[48]	0.543	0.086	0.133	−0.24	−1.33	0.85	
[48]	0.543	0.249	0.383	0.21	−0.88	1.30	
[48]	0.545	0.085	0.131	−0.35	−1.37	0.67	
[48]	0.545	0.302	0.464	0.25	−0.76	1.27	
[50] ^3^	0.589	0.091	0.139	−0.68	−1.90	0.53	
[50] ^3^	0.589	0.179	0.276	0.07	−1.11	1.25	
[50] ^3^	0.589	0.270	0.415	−0.11	−1.29	1.07	
[57]	0.544	0.285	0.437	−0.63	−1.75	0.48	
[57]	0.544	0.427	0.656	−0.32	−1.41	0.78	
[58]	0.513	0.096	0.147	0.58	−0.53	1.69	
[58]	0.513	0.239	0.367	−0.40	−1.50	0.70	
[58]	0.513	0.096	0.147	1.35	0.14	2.55	
[58]	0.513	0.239	0.367	0.00	−1.09	1.09	
**Ross308 (I^2^ = 0.0% P_Q_ = 0.730)**				**0.01**	**−0.17**	**0.18**	
**Combined effect size (I^2^ = 0.0% P_Q_ = 0.850)**				**−0.06**	**−0.20**	**0.08**	

^1^ Without the diluent (glucose, corn starch, CaCO_3_, etc.). ^2^ Without silica. ^3^ SID values calculated from total AA.

**Table 11:** Evaluation of the effect size on body weight gain (BWG) and forest plot of studies with different diet types.

	**Avg. Basal** **Met+Cys, %**	**Supplementation,** **%**					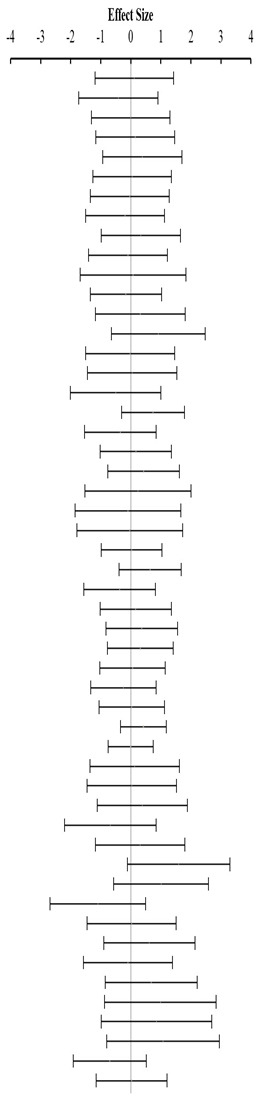
**Study Name/Subgroup Name**		**65 DLM ^1^**	**MHA ^2^**	**Effect Size**	**CI LL**	**CI UL**	
[38]	0.580	0.074	0.114	0.12	−1.19	1.43	
[38]	0.580	0.148	0.228	−0.41	−1.74	0.91	
[38]	0.580	0.222	0.342	0.00	−1.31	1.31	
[38]	0.580	0.296	0.456	0.15	−1.16	1.46	
[38]	0.580	0.370	0.570	0.38	−0.94	1.70	
[38]	0.520	0.062	0.095	0.05	−1.26	1.35	
[38]	0.520	0.124	0.190	−0.03	−1.34	1.28	
[38]	0.520	0.186	0.286	−0.19	−1.50	1.13	
[38]	0.520	0.248	0.381	0.34	−0.98	1.66	
[38]	0.520	0.310	0.476	−0.09	−1.40	1.22	
[39]	0.592	0.236	0.364	0.08	−1.68	1.84	
[43]	0.555	0.223	0.341	−0.16	−1.34	1.02	
[19] ^4^	0.539	0.020	0.030	0.32	−1.17	1.82	
[19] ^4^	0.539	0.039	0.060	0.92	−0.64	2.48	
[19] ^4^	0.539	0.059	0.090	−0.02	−1.50	1.47	
[19] ^4^	0.539	0.078	0.120	0.05	−1.44	1.53	
[19] ^4^	0.539	0.098	0.150	−0.50	−2.00	1.01	
[45] ^4^	0.648	0.180	0.278	0.74	−0.30	1.79	
[46]	0.577	0.092	0.136	−0.34	−1.53	0.85	
[46]	0.577	0.176	0.279	0.17	−1.01	1.36	
[46]	0.577	0.269	0.415	0.43	−0.77	1.63	
[47]	0.540	0.093	0.143	0.24	−1.52	2.00	
[47]	0.540	0.186	0.286	−0.09	−1.85	1.66	
[47]	0.540	0.279	0.429	−0.03	−1.79	1.73	
[48]	0.545	0.085	0.131	0.03	−0.99	1.04	
[48]	0.545	0.302	0.464	0.65	−0.39	1.68	
[50] ^4^	0.589	0.091	0.139	−0.37	−1.56	0.82	
[50] ^4^	0.589	0.179	0.276	0.17	−1.02	1.35	
[50] ^4^	0.589	0.270	0.415	0.37	−0.82	1.56	
[51]	0.540	0.112	0.172	0.32	−0.77	1.42	
[51]	0.540	0.167	0.258	0.06	−1.02	1.15	
[51]	0.540	0.223	0.343	−0.24	−1.33	0.85	
[51]	0.540	0.279	0.429	0.03	−1.06	1.12	
[52]	0.548	0.141	0.218	0.42	−0.34	1.18	
[52]	0.500	0.179	0.275	0.00	−0.75	0.75	
[53] ^4^	0.507	0.315	0.486	0.13	−1.35	1.62	
[53] ^4^	0.507	0.315	0.486	0.03	−1.45	1.52	
[53] ^4^	0.507	0.315	0.486	0.39	−1.11	1.88	
[53] ^4^	0.507	0.315	0.486	−0.68	−2.21	0.85	
[53] ^4^	0.507	0.315	0.486	0.31	−1.18	1.81	
[53] ^4^	0.507	0.315	0.486	1.60	−0.11	3.30	
[53] ^4^	0.507	0.440	0.676	1.01	−0.56	2.59	
[53] ^4^	0.507	0.440	0.676	−1.09	−2.68	0.50	
[53] ^4^	0.507	0.440	0.676	0.03	−1.45	1.51	
[53] ^4^	0.507	0.440	0.676	0.62	−0.90	2.14	
[53] ^4^	0.507	0.440	0.676	−0.09	−1.58	1.39	
[53] ^4^	0.507	0.440	0.676	0.68	−0.84	2.21	
[54]	0.570	0.111	0.170	0.99	−0.87	2.85	
[54]	0.570	0.221	0.340	0.86	−0.98	2.70	
[54]	0.570	0.332	0.511	1.08	−0.80	2.96	
[55]	0.575	0.110	0.165	−0.69	−1.91	0.52	
[55]	0.575	0.210	0.320	0.03	−1.15	1.21	
[56] ^4^	0.527	0.053	0.082	−0.02	−1.78	1.73	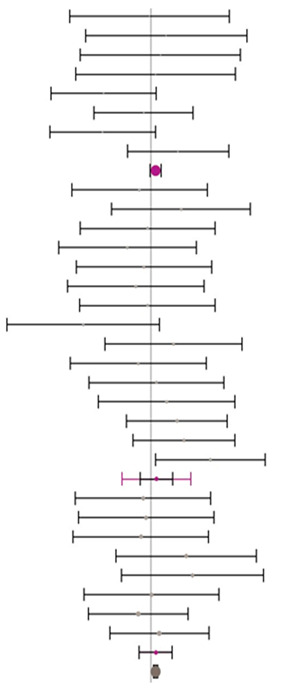
[56] ^4^	0.527	0.107	0.164	0.34	−1.43	2.11	
[56] ^4^	0.527	0.177	0.272	0.22	−1.55	1.98	
[56] ^4^	0.527	0.301	0.462	0.11	−1.65	1.86	
[58]	0.513	0.096	0.147	−1.04	−2.19	0.12	
[58]	0.513	0.239	0.367	−0.16	−1.24	0.93	
[58]	0.513	0.096	0.147	−1.06	−2.22	0.10	
[58]	0.513	0.239	0.367	0.60	−0.51	1.71	
**Corn SBM (I^2^ = 0.0% P_Q_ = 0.861)**				**0.11**	**−0.02**	**0.23**	
[40] ^3^	0.591	0.238	0.366	−0.24	−1.73	1.25	
[42]	0.566	0.255	0.393	0.67	−0.86	2.19	
[35]	0.568	0.020	0.030	−0.07	−1.55	1.41	
[35]	0.568	0.039	0.060	−0.51	−2.02	1.00	
[35]	0.568	0.065	0.100	−0.15	−1.64	1.34	
[35]	0.568	0.098	0.150	−0.33	−1.82	1.17	
[35]	0.568	0.137	0.210	−0.07	−1.56	1.41	
[17]	0.559	0.026	0.040	−1.48	−3.16	0.19	
[17]	0.559	0.052	0.080	0.50	−1.01	2.01	
[17]	0.559	0.078	0.120	−0.28	−1.77	1.22	
[17]	0.559	0.137	0.210	0.13	−1.36	1.61	
[17]	0.559	0.195	0.300	0.35	−1.15	1.85	
[57]	0.544	0.142	0.219	0.58	−0.53	1.68	
[57]	0.544	0.285	0.437	0.73	−0.39	1.85	
[57]	0.544	0.427	0.656	1.31	0.11	2.51	
**Wheat Corn SBM (I^2^ = 23.2% P_Q_ = 0.197)**				**0.14**	**−0.21**	**0.50**	
[37] ^4^	0.648	0.220	0.340	−0.17	−1.65	1.32	
[19]	0.555	0.026	0.040	−0.10	−1.58	1.39	
[19]	0.555	0.052	0.080	−0.21	−1.70	1.27	
[19]	0.555	0.078	0.120	0.78	−0.76	2.32	
[19]	0.555	0.104	0.160	0.92	−0.64	2.48	
[19]	0.555	0.130	0.200	0.02	−1.47	1.50	
[48]	0.543	0.086	0.133	−0.27	−1.37	0.82	
[48]	0.543	0.249	0.383	0.19	−0.90	1.28	
**Wheat SBM (I^2^ = 0.0% P_Q_ = 0.731)**				**0.11**	**−0.26**	**0.48**	
**Combined effect size (I^2^ = 0.0% P_Q_ = 0.823)**				**0.11**	**0.09**	**0.13**	

^1^ Without the diluent (glucose, corn starch, CaCO_3_, etc.). ^2^ Without silica. ^3^ Supplementation or dietary Met+Cys in basal diet recalculated according to AMINOChick^®^ 3.0 software. ^4^ SID values calculated from total AA.

**Table 12:** Evaluation of the effect size on feed conversion ratio (FCR) and forest plot of studies with different diet types.

	**Avg. Basal** **Met+Cys, %**	**Supplementation,** **%**					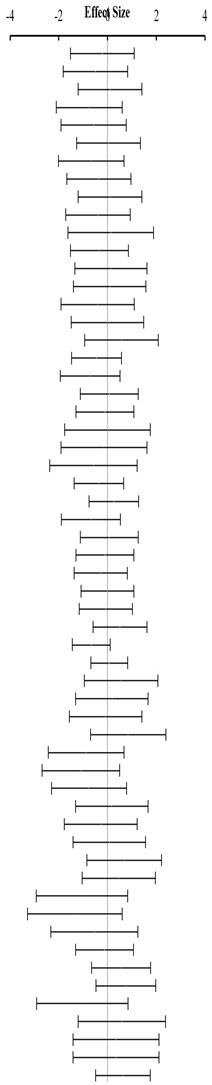
**Study Name/Subgroup Name**		**65 DLM ^1^**	**MHA ^2^**	**Effect Size**	**CI LL**	**CI UL**	
[38]	0.580	0.074	0.114	−0.21	−1.52	1.10	
[38]	0.580	0.148	0.228	−0.50	−1.82	0.83	
[38]	0.580	0.222	0.342	0.11	−1.20	1.42	
[38]	0.580	0.296	0.456	−0.75	−2.10	0.61	
[38]	0.580	0.370	0.570	−0.57	−1.90	0.77	
[38]	0.520	0.062	0.095	0.04	−1.27	1.34	
[38]	0.520	0.124	0.190	−0.67	−2.02	0.67	
[38]	0.520	0.186	0.286	−0.35	−1.67	0.96	
[38]	0.520	0.248	0.381	0.11	−1.20	1.42	
[38]	0.520	0.310	0.476	−0.39	−1.71	0.93	
[39]	0.592	0.236	0.364	0.13	−1.62	1.89	
[43]	0.555	0.223	0.341	−0.33	−1.52	0.86	
[19] ^4^	0.539	0.020	0.030	0.14	−1.35	1.63	
[19] ^4^	0.539	0.039	0.060	0.09	−1.40	1.57	
[19] ^4^	0.539	0.059	0.090	−0.41	−1.90	1.09	
[19] ^4^	0.539	0.078	0.120	0.00	−1.48	1.48	
[19] ^4^	0.539	0.098	0.150	0.57	−0.94	2.09	
[45] ^4^	0.648	0.180	0.278	−0.45	−1.48	0.57	
[46]	0.577	0.092	0.136	−0.71	−1.93	0.51	
[46]	0.577	0.176	0.279	0.07	−1.11	1.25	
[46]	0.577	0.269	0.415	−0.11	−1.29	1.08	
[47]	0.540	0.093	0.143	0.00	−1.76	1.76	
[47]	0.540	0.186	0.286	−0.14	−1.90	1.61	
[47]	0.540	0.279	0.429	−0.58	−2.37	1.21	
[48]	0.545	0.085	0.131	−0.35	−1.37	0.67	
[48]	0.545	0.302	0.464	0.25	−0.76	1.27	
[50] ^4^	0.589	0.091	0.139	−0.68	−1.90	0.53	
[50] ^4^	0.589	0.179	0.276	0.07	−1.11	1.25	
[50] ^4^	0.589	0.270	0.415	−0.11	−1.29	1.07	
[51]	0.540	0.112	0.172	−0.28	−1.37	0.81	
[51]	0.540	0.167	0.258	0.00	−1.09	1.09	
[51]	0.540	0.223	0.343	−0.07	−1.16	1.02	
[51]	0.540	0.279	0.429	0.51	−0.60	1.61	
[52]	0.548	0.141	0.218	−0.66	−1.44	0.11	
[52]	0.500	0.179	0.275	0.07	−0.69	0.82	
[53] ^4^	0.507	0.315	0.486	0.55	−0.96	2.07	
[53] ^4^	0.507	0.315	0.486	0.18	−1.31	1.66	
[53] ^4^	0.507	0.315	0.486	−0.08	−1.57	1.40	
[53] ^4^	0.507	0.315	0.486	0.85	−0.70	2.39	
[53] ^4^	0.507	0.315	0.486	−0.87	−2.43	0.68	
[53] ^4^	0.507	0.315	0.486	−1.09	−2.68	0.50	
[53] ^4^	0.507	0.440	0.676	−0.76	−2.29	0.78	
[53] ^4^	0.507	0.440	0.676	0.17	−1.31	1.66	
[53] ^4^	0.507	0.440	0.676	−0.28	−1.77	1.21	
[53] ^4^	0.507	0.440	0.676	0.07	−1.41	1.56	
[53] ^4^	0.507	0.440	0.676	0.69	−0.84	2.22	
[53] ^4^	0.507	0.440	0.676	0.46	−1.04	1.96	
[54]	0.570	0.111	0.170	−1.05	−2.92	0.82	
[54]	0.570	0.221	0.340	−1.34	−3.29	0.60	
[54]	0.570	0.332	0.511	−0.54	−2.33	1.25	
[55]	0.575	0.110	0.165	−0.12	−1.31	1.06	
[55]	0.575	0.210	0.320	0.56	−0.65	1.76	
[55]	0.575	0.320	0.485	0.75	−0.47	1.97	
[56] ^4^	0.527	0.053	0.082	−1.03	−2.90	0.84	
[56] ^4^	0.527	0.107	0.164	0.59	−1.20	2.39	
[56] ^4^	0.527	0.177	0.272	0.35	−1.42	2.12	
[56] ^4^	0.527	0.301	0.462	0.35	−1.42	2.12	
[58]	0.513	0.096	0.147	0.63	−0.48	1.75	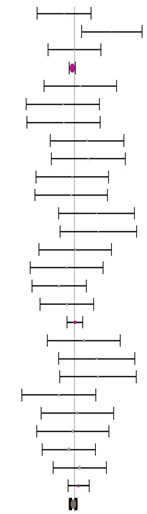
[58]	0.513	0.239	0.367	−0.43	−1.53	0.67	
[58]	0.513	0.096	0.147	1.53	0.29	2.77	
[58]	0.513	0.239	0.367	0.00	−1.09	1.09	
**Corn SBM (I^2^ = 0.0% P_Q_ = 0.662)**				**−0.09**	**−0.22**	**0.04**	
[40] ^3^	0.591	0.238	0.366	0.24	−1.25	1.73	
[42]	0.566	0.255	0.393	−0.48	−1.98	1.03	
[35]	0.568	0.020	0.030	−0.45	−1.95	1.06	
[35]	0.568	0.039	0.060	0.51	−0.99	2.02	
[35]	0.568	0.065	0.100	0.56	−0.95	2.08	
[35]	0.568	0.098	0.150	−0.10	−1.58	1.39	
[35]	0.568	0.137	0.210	−0.14	−1.62	1.35	
[17]	0.559	0.052	0.080	0.90	−0.66	2.45	
[17]	0.559	0.078	0.120	0.97	−0.60	2.54	
[17]	0.559	0.137	0.210	0.02	−1.46	1.51	
[17]	0.559	0.195	0.300	−0.32	−1.82	1.17	
[57]	0.544	0.285	0.437	−0.63	−1.75	0.48	
[57]	0.544	0.427	0.656	−0.32	−1.41	0.78	
**Wheat Corn SBM (I^2^ = 0.0% P_Q_ = 0.581)**				**0.01**	**−0.31**	**0.33**	
[37] ^4^	0.648	0.220	0.340	0.38	−1.12	1.87	
[19]	0.555	0.026	0.040	0.91	−0.65	2.47	
[19]	0.555	0.052	0.080	0.96	−0.61	2.52	
[19]	0.555	0.078	0.120	−0.64	−2.17	0.88	
[19]	0.555	0.104	0.160	0.11	−1.37	1.60	
[19]	0.555	0.130	0.200	−0.07	−1.55	1.42	
[48]	0.543	0.086	0.133	−0.24	−1.33	0.85	
[48]	0.543	0.249	0.383	0.21	−0.88	1.30	
**Wheat SBM (I^2^ = 0.0% P_Q_ = 0.527)**				**0.17**	**−0.27**	**0.60**	
**Combined effect size (I^2^ = 0.0% P_Q_ = 0.722)**				**−0.05**	**−0.16**	**0.06**	

^1^ Without the diluent (glucose, corn starch, CaCO_3_, etc.). ^2^ Without silica. ^3^ Supplementation or dietary Met+Cys in basal diet recalculated according to AMINOChick^®^ 3.0 software. ^4^ SID values calculated from total.

## Data Availability

Data available on request. While most of the data were obtained from peer-reviewed scientific papers, others were taken from the grey literature. While those are available in principle, a direct request to the corresponding author would speed this process up.
